# Si-Wu-Tang Targets Microbiota Homeostasis and Intestinal Mucosal Barriers to Provide Protection Against MASLD by Favoring *P. goldsteinii*-like Taxa Colonization

**DOI:** 10.3390/ph19030400

**Published:** 2026-02-28

**Authors:** Xiaoyong Xue, Fukun Zhang, Hong Wang, Mengyu Guo, Wenqing Qin, Yun Yang, Zixuan Huo, Xin Li, Qi Han, Xiaojiaoyang Li

**Affiliations:** 1 School of Life Sciences, Beijing University of Chinese Medicine, 11 Bei San Huan Dong Lu, Beijing 100029, China; xuexiaoyonga@163.com (X.X.); zhangfukun0711@163.com (F.Z.); wangh_iris@163.com (H.W.); 15069125570@163.com (M.G.); yy18810628253@163.com (Y.Y.); hzx13081839882@163.com (Z.H.); 2 School of Chinese Materia Medica, Beijing University of Chinese Medicine, 11 Bei San Huan Dong Lu, Beijing 100029, China; 16650336137@163.com (W.Q.); lixinbjzyydx@163.com (X.L.)

**Keywords:** Si-Wu-Tang, MASLD, gut–liver axis, gut microbiome, alcohol

## Abstract

**Objective:** This study examined the pharmacological mechanisms of the therapeutic benefits of SWT to MASLD via regulating the gut–liver axis. **Methods:** The components of SWT were analyzed by liquid chromatograph mass spectrometer (LC-MS). After establishing an MCD-induced MASLD mice model, we invested the protective mechanism of SWT through 16S rRNA sequencing combined with molecular biological experiments. After eliminating the intestinal microbiota through an antibiotic cocktail experiment, we identified the key microbiota by which SWT improves MASLD. **Results:** SWT markedly reduced MASLD injury by alleviating intestinal inflammation and restoring the intestinal mucosal barrier, which could be reversed following alcohol exposure. Additionally, SWT altered the intestinal flora of MASLD mice, significantly raising the relative abundance of *Parabacteroides goldsteinii*-like taxa, while alcohol caused the destruction of *P. goldsteinii*-like-taxa-centered probiotic habitats and a proliferation of pathogenic bacteria, especially *Bacteroides intestinalis*-like taxa. After the elimination of intestinal flora, the anti-MASLD effect of SWT was lost. Moreover, the supplement of *P. goldsteinii* could significantly ameliorate liver damage caused by an MCD diet, functioning similarly to SWT. However, the liver-protective effect of SWT was suppressed following the administration of *B. intestinalis*. **Conclusions:** SWT ameliorates MCD diet-induced MASLD via modulating intestinal microbiota homeostasis and restoring intestinal mucosal barriers. Given that *P. goldsteinii* is effective for treating MASLD, it provides insights into new therapeutic strategies.

## 1. Introduction

Metabolic dysfunction-associated steatotic liver disease (MASLD) is a chronic metabolic disease that affects approximately 38% of adults worldwide. MASLD can potentially lead to more severe pathologies, such as non-alcoholic steatohepatitis (NASH), cirrhosis and, ultimately, hepatocellular carcinoma (HCC) [1,2]. Resmetirom being the only FDA-approved treatment option, coupled with the rising incidence rate of MASLD, fails to meet the clinical requirements of every patient [3]. Furthermore, although MASLD primarily affects the liver, the interconnectedness of various organs implies that diseases impacting other organs can also influence the occurrence and progression of MASLD; for instance, inflammatory bowel disease (IBD) is closely associated with MASLD. Research indicated that lean patients with IBD were not only more susceptible to developing MASLD, but they also tended to experience exacerbated liver damage due to this condition, which significantly increased their mortality risk [4,5,6]. Consequently, monitoring dietary habits and improving the intestinal microenvironment may become effective strategies to limit the occurrence and progression of MASLD.

The gut–liver axis establishes a connection between the liver and the intestine via the intestinal microbiota and its associated metabolites. This interaction is influenced by signals generated from dietary, genetic and environmental factors. Recent research has suggested that the dysfunction in the hepato-enteric axis plays a critical role in the development and advancement of MASLD [7]. Chronic inflammatory diseases affecting the gastrointestinal tract are often linked to an imbalance in gut microbiota, which heightens the inflammatory reaction and plays a role in the onset of MASLD. Studies have identified various pro-inflammatory cytokines in the intestines of patients with IBD, such as interferon-alpha (IFN-α) and interleukin-6 (IL-6). These cytokines can weaken the intestinal mucosal barrier, enhance intestinal permeability, and facilitate the inappropriate translocation of gut microbiota [8]. As increasing quantities of bacteria and relevant metabolites traverse the portal vein into the liver, the liver undergoes inflammation, oxidative stress and lipid accumulation, thereby eventually resulting in the progression of MASLD [9,10]. Concurrently, patients with MASLD often exhibit disturbances in intestinal flora and higher changes in intestinal diseases [11]. Recent research has shown that mice fed a high-fat diet exhibiting significant proliferation of Clostridium difficile within the gut, elevating secondary bile acids (BAs) in the ileum and resulting in intestinal flora disturbance. This disruption hindered the normal enterohepatic circulation and inhibited the activation of the farnesoid X receptor (FXR) in the liver, thereby exacerbating the damage associated with MASLD [5]. In addition, chronic excessive alcohol consumption not only contributed to elevated intestinal mucosa permeability and immune system disruption, but also led to poorer prognosis and elevated mortality rates among patients with MASLD [12,13,14,15]. Consequently, regulating the enterohepatic axis and restoring intestinal microbiota homeostasis emerges as an effective strategy for the treatment of MASLD.

Compared to Western medicine, traditional Chinese medicine (TCM) frequently yields more effective treatment results for chronic conditions by influencing several organs while keeping toxicity levels lower. SWT, a classic TCM formula consisting of *Paeonia lactiflora* Pall. (Baishao), *Rehmannia glutinosa* (Gaertn.) DC. (Shudihuang), *Ligusticum striatum* DC (Chuanxiong) and *Angelica sinensis* (Oliv.) Diels (Danggui) has recently been reported to be used for the treatment of hepatobiliary diseases [16,17,18]. Our previous studies have demonstrated that SWT restored the liver’s immune microenvironment and alleviated bile duct ligation (BDL)-induced cholestatic liver injury by regulating multiple immune cells, especially for macrophages, neutrophils and CD8 + T cells, and promoting activated hepatic stellate cells apoptosis through the activation of the FAS pathway [19]. Recently, we found that SWT has a protective function in MASLD, induced by a methionine–choline deficiency (MCD) diet, which is mediated by the suppression of ACSL4-driven arachidonic acid metabolism and ferroptosis in hepatocytes [20]. In another investigation, we discovered that SWT enhances liver fibrosis by restoring the intestinal mucosal barrier [21]. Therefore, we propose the scientific hypothesis that SWT modulates microbiota balance and intestinal mucosal integrity via the liver–gut axis to improve MASLD.

To explore whether SWT has a regulatory effect on the intestinal flora disorder in MASLD mice and to clarify the key flora therein, we conducted 16S rRNA sequencing. Further, combined with the analysis of 16S rRNA sequencing and microbiota functional correlation analysis, we found that SWT improved MASLD injury by correcting the unbalanced intestinal flora homeostasis and restoring the intestinal mucosal barrier. Interestingly, the anti-MASLD efficacy of SWT was diminished in mice that were subjected to long-term alcohol consumption, thereby demonstrating that alcohol is a significant risk factor that adversely affects the efficacy of traditional Chinese medicine. Furthermore, after depleting the intestinal flora using antibiotics, we observed that the efficacy of SWT in improving MASLD was diminished. In contrast, the supplementation of *P. goldsteinii* significantly enhanced MASLD. This indicates that *P. goldsteinii* is a crucial microbiota for the effectiveness of SWT in ameliorating MASLD. Our results not only provide experimental evidence for the molecular mechanism of SWT in improving MASLD, based on the liver–gut axis, but also highlight precautions that are necessary to ensure the clinical efficacy of SWT.

## 2. Results

### 2.1. Chemical Characterization of SWT

The chemical components of SWT were identified and preliminarily characterized using LC-MS. In the total ion current chromatograms in both positive and negative ionization modes, we separated 22 representative ingredients from *Ligusticum chuanxiong* Hort., *Angelica sinensis* (Oliv.) Diels, *Paeonia lactiflora* Pall. and *Rehmannia glutinosa* Libosch (Figure S1A,B). Meanwhile, four major ingredients including albiflorin, 3-*N*-butyl-4,5-dihydrophthalide, manninotriose and isochlorogenic acid A were selected as representatives to evaluate the quality of SWT. The precursor and product ion spectra of these components were shown in Figure S1C–F.

### 2.2. SWT Significantly Alleviates Liver Injury in MCD Diet-Induced Mouse Model

**Figure 1:**
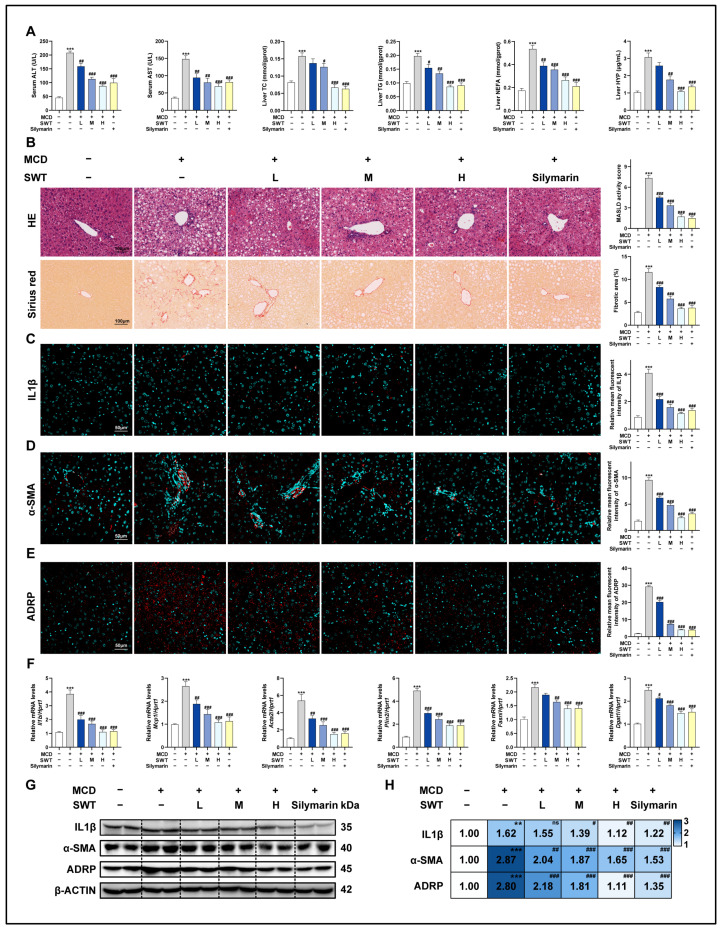
SWT protects against MCD diet-induced liver injury in mice. (**A**) Serum ALT/AST and hepatic TC, TG, NEFA, and HYP. (**B**) H&E and Sirius red staining of liver sections (scale bar, 100 μm). (**C**–**E**) IF staining of IL1β (**C**), α-SMA (**D**), and ADRP (**E**) in liver sections with quantification (scale bar, 50 μm). (**F**) Hepatic mRNA levels of *Il1b*, *Mcp1*, *Acta2*, *Plin2*, *Fasn*, and *Dgat1*. (**G**,**H**) Protein levels of IL1β, α-SMA, and ADRP in liver sections. Statistical significance: ns, no statistical differences vs. MCD group; ** *p* < 0.01, *** *p* < 0.001 vs. control group; ^#^ *p* < 0.05, ^##^ *p* < 0.01, ^###^ *p* < 0.001 vs. MCD group. Data were presented as means ± SEM. One-way ANOVA with Tukey’s post hoc tests (*n* = 6).

We assessed the expression of various liver damage markers in mouse serum in order to investigate the protective effects of SWT on the murine model of MASLD. The results showed that SWT markedly and dose-dependently reduced the increased expression of the alanine aminotransferase (ALT), aspartate aminotransferase (AST), total cholesterol (TC), triglyceride (TG), hepatic non-esterified fatty acids (NEFA), and hepatic hydroxyproline (HYP) induced by the MCD diet (Figure 1A). Additionally, we performed H&E and Sirius red staining to investigate histological changes in different groups (Figure 1B). In comparison with the control group, the MCD diet caused disrupted structures in the hepatic area, accompanied with numerous infiltrating immune cells and massive collagen deposition, but were alleviated by SWT administration. In addition, the immunofluorescence staining (Figure 1C–E and Figure S2A–C) and Western blotting (Figure 1G,H) results showed significantly elevated expression of interleukin 1 beta (IL1β), α-smooth muscle actin (α-SMA), and adipose differentiation-related protein (ADRP) around hepatic portal areas in the MCD group, which were markedly reduced with SWT administration. Consistently, we found that the markers of inflammatory response (IL1β; monocyte chemoattractant protein 1, MCP1), liver fibrosis (actin alpha 2, ACTA2), and lipid accumulation-associated (perilipin 2, PLIN2; fatty acid synthase, FASN; diacylglycerol acyltransferase 1, DGAT1) in liver samples were significantly elevated in the MCD groups at mRNA level using qPCR, which were all significantly reduced after SWT treatment (Figure 1F). These findings showed that SWT has hepatoprotective effects on liver damage, induced by the MCD diet.

### 2.3. The Liver-Protective Effect of SWT Is Suppressed Following Alcohol Exposure in MCD Diet-Induced Mouse Model

**Figure 2:**
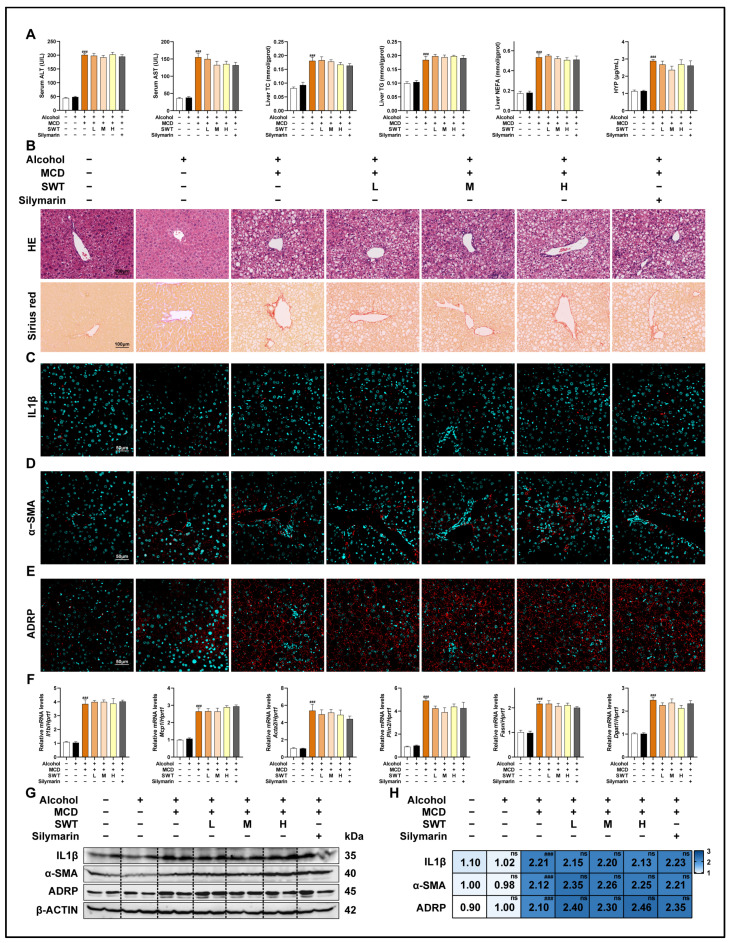
Alcohol compromises SWT-mediated hepatoprotection in MASLD. (**A**) Serum ALT/AST and hepatic TC, TG, NEFA, and HYP. (**B**) H&E and Sirius red staining of liver sections (scale bar, 100 μm). (**C**–**E**) IF staining of IL1β (**C**), α-SMA (**D**), and ADRP (**E**) in liver sections (scale bar, 50 μm). (**F**) Hepatic mRNA levels of *Il1b*, *Mcp1*, *Acta2*, *Plin2*, *Fasn*, and *Dgat1*. (**G**,**H**) Protein levels of IL1β, α-SMA, and ADRP in liver sections. Statistical significance: ^###^ *p* < 0.001 vs. MCD group; ns, no statistical differences among MCD + alcohol, MCD + alcohol + SWT low dose (L), MCD + alcohol + SWT medium dose (M), MCD + alcohol + SWT high dose (H), and MCD + alcohol + silymarin group. Data were presented as means ± SEM. One-way ANOVA with Tukey’s post hoc tests (*n* = 6).

After confirming that SWT markedly alleviated the MCD diet-induced liver injury and improved inflammatory, fibrotic, and lipid-accumulation phenotypes, we further investigated whether its protective efficacy could be affected by alcohol exposure, a common clinical confounder. Given that alcohol can alter the activity of hepatic drug-metabolizing enzymes and may consequently influence the systemic exposure and therapeutic efficacy of traditional herbal medicines containing volatile constituents, we incorporated an alcohol intervention into the MASLD model to evaluate whether alcohol would modify the effects of SWT. Specifically, 30% alcohol was administered by gavage to both healthy C57BL/6J mice and MCD diet-fed mice. Alcohol alone did not cause obvious abnormalities in serum biochemical parameters; however, when superimposed on the MCD model, alcohol markedly exacerbated the liver injury phenotype (Figure 2A). More importantly, under alcohol exposure, the therapeutic effects of SWT on these abnormal indices were almost completely abolished: liver injury-related parameters in the MCD + alcohol + SWT group remained elevated and were comparable to those in the MCD + alcohol group, indicating that alcohol weakened or even offset the hepatoprotective effects of SWT (Figure 2A). Histological evidence was consistent with the biochemical findings (Figure 2B). At the molecular and cellular levels, immunofluorescence staining and immunoblotting further demonstrated that, in the presence of alcohol, SWT was unable to effectively reduce the MCD-induced upregulation of IL1β, α-SMA, and ADRP (Figure 2C–E,G,H and Figure S3A–C). Consistently, qPCR analyses showed that the expression of inflammation-related transcripts (IL1β and MCP1), the fibrosis marker ACTA2, and lipid-accumulation-associated genes (PLIN2, FASN, and DGAT1) remained high in the MCD + alcohol + SWT group (Figure 2F). Collectively, these results indicate that alcohol not only aggravates MCD-induced liver injury but also substantially impairs the protective effects of SWT, underscoring that alcohol exposure should be considered a critical confounding factor when evaluating and applying SWT for MASLD treatment.

### 2.4. Alcohol Weakens the Improvement of MCD Diet-Induced Intestinal Mucosal Barrier Damage by SWT

**Figure 3:**
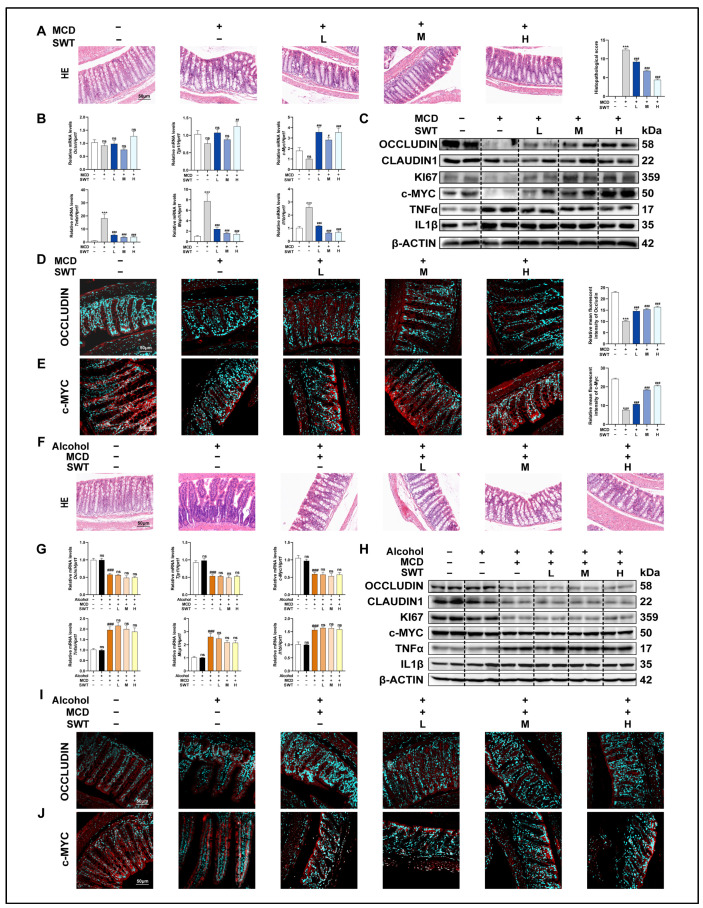
Alcohol weakens the intestinal protective effect of SWT in MASLD. (**A**) H&E staining of intestine tissues (scale bar, 50 μm). (**B**) Intestinal mRNA levels of *Tnfα*, *Il1b*, *Mcp1*, *c-Myc*, *Ocln*, and *Tjp1*. (**C**) Protein levels of OCCLUDIN, CLAUDIN-1, KI67, c-MYC, TNFα, and IL1β in intestine. (**D**,**E**) IF staining of OCCLUDIN and c-MYC in intestine with quantification (scale bar, 50 μm). (**F**) H&E staining under alcohol exposure (scale bar, 50 μm). (**G**) Intestinal mRNA levels as in (**B**). (**H**) Protein levels as in (**C**). (**I**,**J**) IF staining of OCCLUDIN and c-MYC in intestine with quantification (scale bar, 50 μm). Statistical significance: *** *p* < 0.001 vs. control group; ^#^ *p* < 0.05, ^##^ *p* < 0.01, ^###^*p* < 0.001, vs. MCD or MCD + alcohol group; ns, no statistical differences among control and alcohol group. Data were presented as means ± SEM. One-way ANOVA with Tukey’s post hoc tests (*n* = 6).

Given that the integrity of the intestinal mucosal barrier is considered a critical prerequisite for SWT-mediated hepatoprotection, we shifted our focus to the architecture of the ileum and the molecular network associated with barrier function. At the morphological level, H&E staining of the ileum showed that the MCD diet induced villus disruption accompanied by inflammatory changes, whereas SWT treatment progressively ameliorated these inflammatory features and restored villus integrity in a dose-dependent manner (Figure 3A). On this basis, we further evaluated the “inflammation-driven barrier disruption” axis: upon MCD challenge, inflammation-related genes such as TNFα, IL1β, and MCP1 were markedly upregulated; SWT significantly suppressed their induction at the transcript level and concomitantly reduced TNFα and IL1β protein expression (Figure 3B,C), indicating that SWT effectively alleviates the local intestinal inflammatory burden. Notably, the effects of SWT were not limited to anti-inflammatory regulation but were also accompanied by enhanced signals related to barrier repair and epithelial renewal. Compared with the MCD group, SWT significantly increased the expression of tight junction-associated proteins (OCCLUDIN/OCLN, CLAUDIN-1/CLDN1, and TJP1) and epithelial proliferation-related markers (c-MYC and KI67) (Figure 3B,C). Immunofluorescence staining further validated, in a spatial position manner, the differential expression of OCCLUDIN, c-MYC, and IL1β among groups (Figure 3D,E, Figures S4A,B and S5A). Collectively, these results suggest that SWT simultaneously attenuates inflammatory responses while strengthening mucosal barrier function and epithelial renewal in the MCD model. We next used alcohol exposure as an exogenous perturbation to test whether the above “barrier–proliferation” improvement is vulnerable to disruption. Histological examination revealed that both the MCD + alcohol and MCD + alcohol + SWT groups still exhibited prominent villus injury accompanied by extensive immune cell infiltration (Figure 3F), suggesting that alcohol aggravates intestinal damage and limits SWT-mediated structural repair. Molecular evidence supported this observation: compared with controls, the MCD + alcohol group showed significantly elevated inflammatory mediators (TNFα, MCP1, and IL1β) but markedly reduced the expression of barrier- and proliferation-related factors (OCCLUDIN, TJP1, and c-MYC) (Figure 3G,H); moreover, in the presence of alcohol, the extent to which SWT corrected these alterations was substantially diminished. Immunofluorescence staining likewise corroborated the group-wise changes in Occludin, c-MYC, and IL1β (Figure 3I,J, Figures S4C,D and S5B). In summary, SWT exerts dual effects in MCD-induced intestinal injury by suppressing inflammation while promoting epithelial proliferation and tight-junction reconstitution; excessive alcohol consumption compromises this reparative process, thereby hindering the effective restoration of the mucosal barrier and exacerbating MCD-associated intestinal inflammatory phenotypes.

### 2.5. SWT and Alcohol Alter the Gut Microbiota in MCD Diet-Induced Mice

**Figure 4:**
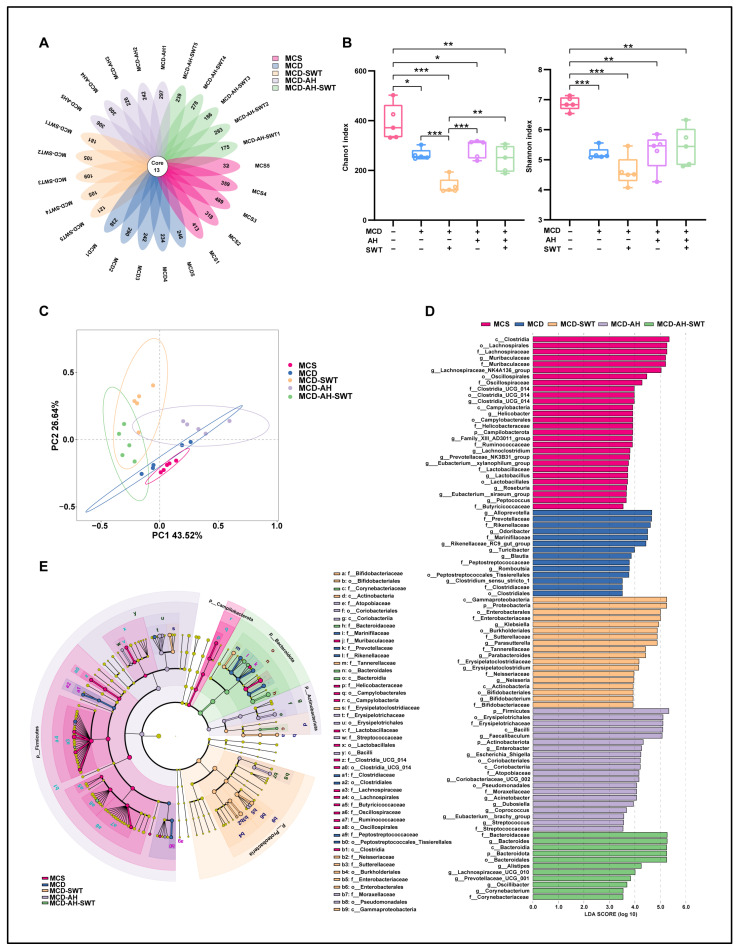
SWT and alcohol reshape gut microbiota in MCD-induced MASLD mice. (**A**) ASV Venn diagram. (**B**) Alpha diversity (Chao1 and Shannon). (**C**) PCoA of beta diversity. (**D**,**E**) Differentially abundant taxa identified by LEfSe (LDA ≥ 3.0). Statistical significance: * *p* < 0.05, ** *p* < 0.01, *** *p* < 0.001 vs. control group; Data were presented as means ± SEM. One-way ANOVA with Tukey’s post hoc tests (*n* = 5).

To further explore whether the mechanisms linking the therapeutic properties of SWT to the regulation of the gut–liver axis is connected to the gut microbiota, we extracted bacterial DNA from intestinal contents and analyzed it through 16S rRNA sequencing. Notably, both SWT administration and alcohol exposure significantly altered the diversity of the gut microbiota in MCD diet-induced mice. As illustrated by the Venn diagram analysis of the Amplicon sequence variant (ASV) (Figure 4A), there was an obvious decline in overall ASV in the MCD + SWT group when compared to the MCD group. Furthermore, the ASV was significantly increased in the MCD + alcohol + SWT group when compared to the MCD + SWT group. We conducted further α and β diversity analyses to evaluate the differences in gut microbiota diversity in different groups. The community richness (Chao1) and the diversity index (Shannon) were used to reflect α diversity between groups. Compared to the control group, MCD exposure reduced the Chao1 index, and this reduction was even more pronounced following SWT treatment. However, the exposure of alcohol led to a significant increase in the Chao1 index in both the MCD + alcohol and MCD + alcohol + SWT groups (Figure 4B, left panel). The Shannon index showed a similar trend (Figure 4B, right panel). Additionally, principal co-ordinates analysis (PCoA) was conducted to assess β diversity, revealing distinct clustering of gut microbiota distribution in five groups (Figure 4C). We further analyzed changes in gut microbiota at different taxonomic levels, based on analysis of the 16S rRNA gene sequencing. The linear discriminant analysis (LDA) histogram (LDA ≥ 2.0) and LDA Effect Size (LEfSe) cladograms further indicated that 91 bacterial clades demonstrated biological and statistical consistency from the phylum to the genus level (Figure 4D,E). Collectively, SWT and alcohol altered the gut microbiota in MCD diet-induced mice.

### 2.6. Probiotics Dominated by P. goldsteinii-like Taxa Display Correlation with the Protective Effects of SWT on MCD Diet-Induced Liver Injury and Intestinal Mucosal Barrier

**Figure 5:**
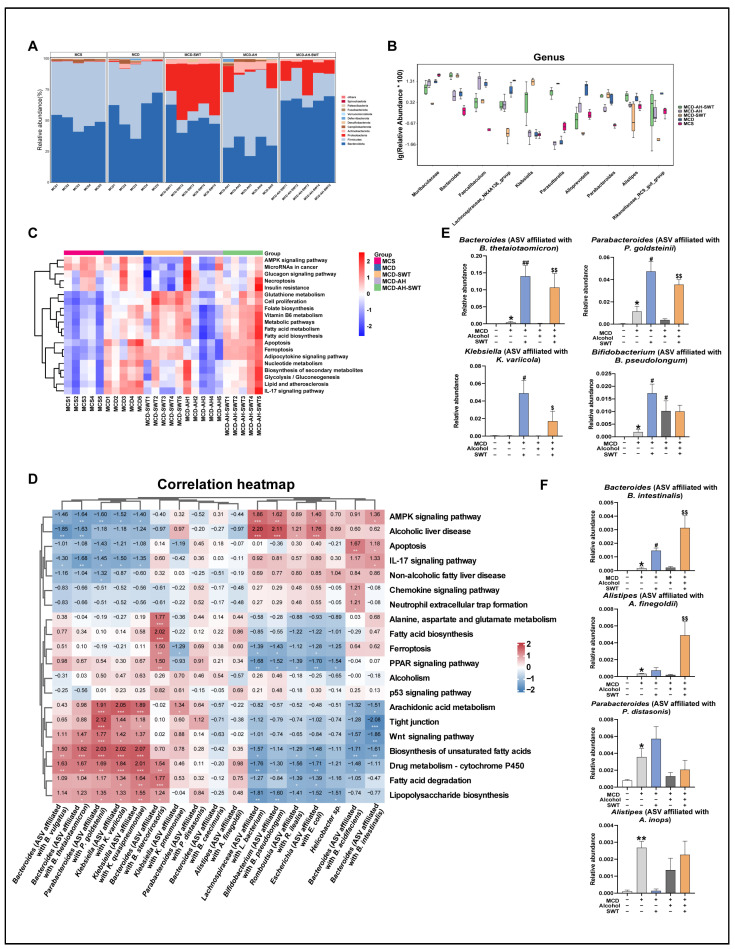
Effects of SWT and alcohol on MASLD-mediated disturbance of gut microbiota and their associated biological functions. (**A**) Phylum-level composition. (**B**) Top 10 species-level taxa. (**C**) The analysis of KEGG pathways in different groups. (**D**) Correlation between predicted KEGG pathways and species-level taxa. (**E**) Relative abundance of probiotics, including *Bacteroides thetaiotaomicron*-like taxa, *Parabacteroides goldsteinii*-like taxa, *Klebsiella variicola*-like taxa and *Bifidobacterium pseudolongum*-like taxa. (**F**) Relative abundance of pathogens, including *Parabacteroides distasonis*-like taxa, *Alistipes finegoldii*-like taxa, *Bacteroides intestinalis*-like taxa, and *Alistipes inops*-like taxa. Statistical significance: * *p* < 0.05, ** *p* < 0.01, *** *p* < 0.001 vs. control group; ^#^ *p* < 0.05, ^##^ *p* < 0.01 vs. MCD group; ^$^ *p* < 0.05, ^$$^ *p* < 0.01 vs. MCD + SWT group. Data were presented as means ± SEM. One-way ANOVA with Tukey’s post hoc tests (*n* = 5).

To explore differences in microbial composition among the groups, we conducted a columnar analysis of abundance data at Figure 5A and Figure S6A,B. We found that SWT and alcohol altered the intestinal microflora structure at the phylum, class, order, family, and genus levels in MCD diet-induced mice. Based on statistical analysis at various taxonomic levels, we identified the top 10 species with the most significant abundance differences for relative abundance boxplot analysis, comparing abundance within and between groups, as shown in Figure 5B.

KEGG analysis, a knowledge base for systematic analysis of gene functions [22], revealed that SWT upregulated lipid metabolism-related pathways, while it downregulated the inflammation and glucose-metabolism-associated pathways (Figure 5C and Figure S6C,D). Tight junctions and the Wnt signaling pathway are involved in maintaining intestinal barrier repair and tissue homeostasis. To further clarify the key bacteria of SWT in improving MASLD, we selected the top 15 bacteria with the most significant abundance differences at the species level in different groups (Figure 5B), and analyzed the correlation between these bacteria and biological functions (Figure 5D and Figure S6E,F). According to the findings, a number of bacteria linked to intestinal inflammation or barrier disturbance, such as beneficial bacteria *Parabacteroides goldsteinii*-like taxa (due to the intrinsic limitations of partial 16S rRNA sequencing for reliable species-level identification), exhibited a significant positive correlation with tight junctions and the Wnt signaling pathway. Conversely, microbiota displayed a negative correlation with the protective processes, including pathogenic bacteria *Bacteroides intestinalis*-like taxa. Combined with the most significant abundance differences at various taxonomic levels and KEGG analysis, the effects of SWT and alcohol on MCD diet-induced liver injury and intestinal mucosal barrier might be mediated by *P. goldsteinii*-like taxa and *B. intestinalis*-like taxa. Furthermore, *P. goldsteinii*-like taxa exhibited a significant negative correlation with alcoholic liver disease as well as non-alcoholic fatty liver disease, while *B. intestinalis*-like taxa displayed a contrary correlation. Additionally, we found that SWT might have altered the abundance of four probiotics (*Bacteroides thetaiotaomicron*-like taxa, *Parabacteroides goldsteinii*-like taxa, *Klebsiella variicola*-like taxa, and *Bifidobacterium pseudolongum*-like taxa) and four pathogenic bacteria (*Parabacteroides distasonis*-like taxa, *Alistipes finegoldii*-like taxa, *Bacteroides intestinalis*-like taxa, and *Alistipes inops*-like taxa) in MCD diet-induced mice (Figure 5E,F). However, under SWT treatment, alcohol exposure impaired the increased relative abundance of *P. goldsteinii*-like-taxa-centered probiotics and caused a proliferation of the pathogenic bacteria, especially *B. intestinalis*-like taxa. Taken together, we speculated that: (1) SWT promoted the competitive colonization of *P. goldsteinii*-like taxa in the gut, which mediated its protective benefits against intestinal mucosal barrier and liver damage caused by the MCD diet. (2) Alcohol reversed this therapeutic via decreasing the relative abundance of *P. goldsteinii*-like-taxa-dominated probiotics and a proliferation of pathogenic bacteria, especially *B. intestinalis*-like taxa.

### 2.7. P. goldsteinii Ameliorates Liver Damage Caused by MCD Diet, Functioning Similarly to SWT

**Figure 6:**
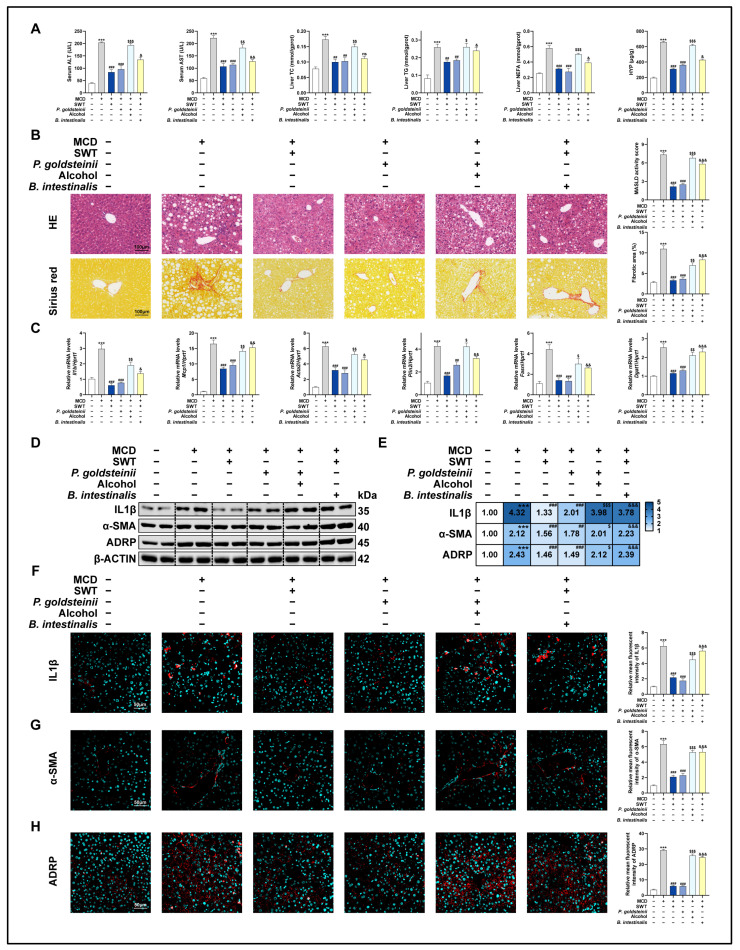
*P. goldsteinii* reduced MCD diet-induced liver injury in mice. (**A**) Serum ALT/AST and hepatic TC, TG, NEFA, HYP. (**B**) Hepatic H&E and Sirius red staining (Scale bar, 100 μm). (**C**) Hepatic mRNA levels of *Il1b*, *Mcp1*, *Acta2*, *Plin2*, *Fasn*, and *Dgat1.* (**D**,**E**) Protein levels of IL1β, α-SMA, and ADRP in liver sections. (**F**–**H**) IF staining of (**F**) IL1β, (**G**) α-SMA, and (**H**) ADRP in liver sections with quantification (Scale bar, 50 μm). Statistical significance: *** *p* < 0.001 vs. control group; ^##^ *p* < 0.01, ^###^ *p* < 0.001 vs. MCD group; ^$^ *p* < 0.05, ^$$^ *p* < 0.01, ^$$$^ *p* < 0.001 vs. MCD + *P. goldsteinii* group; and ^&^ *p* < 0.05, ^&&^ *p* < 0.01, ^&&&^ *p* < 0.001 vs. MCD + SWT group. Data were presented as means ± SEM. One-way ANOVA with Tukey’s post hoc tests (*n* = 6).

To verify the aforementioned conjecture, we initially demonstrated the promoting effect of SWT on the growth and proliferation of *P. goldsteinii* through analyses of growth curves and biofilm formation (Figure S7A,B). We further investigated the effects of the administration of *P. goldsteinii* or *B. intestinalis* on liver damage in MCD diet-induced mice. As expected, the elevated serum levels of ALT, AST, TC, TG, NEFA, and HYP in MCD diet-induced mice were all reduced by the single administration of *P. goldsteinii*, which was similar to that of SWT treatment (Figure 6A). However, these indicators were still at high levels in the MCD + *P. goldsteinii* + alcohol group and the MCD + SWT + *B. intestinalis* group. H&E and Sirius red staining further revealed that *P. goldsteinii* or SWT significantly alleviated inflammatory infiltration and relieved collagen fiber deposition in the livers of MCD diet-induced mice, which could be reversed by supplementing with alcohol or *B. intestinalis*, respectively (Figure 6B). Similar results were observed in the expression of inflammatory cytokines (IL1β and MCP1), the liver fibrosis marker (ACTA2) and lipid accumulation-related factors (PLIN2, FASN, and DGAT1) (Figure 6C). Additionally, immunofluorescence staining also confirmed that *P. goldsteinii* or SWT treatment could reduce the expression of IL1β, α-SMA and ADRP, while co-administration of *P. goldsteinii* and alcohol or SWT and *B. intestinalis* reversed these hepatoprotective effects (Figure 6D–H and Figure S8). These results indicated that: (1) *P. goldsteinii* could significantly ameliorate liver damage caused by the MCD diet, functioning similarly to SWT. (2) The liver-protective effects of *P. goldsteinii* were suppressed following alcohol exposure. (3) *B. intestinalis* could impair the hepatoprotective effects of SWT.

### 2.8. P. goldsteinii Mediates the Therapeutic Potential of SWT on Intestinal Homeostasis and Gut–Liver Axis in MCD Diet

**Figure 7:**
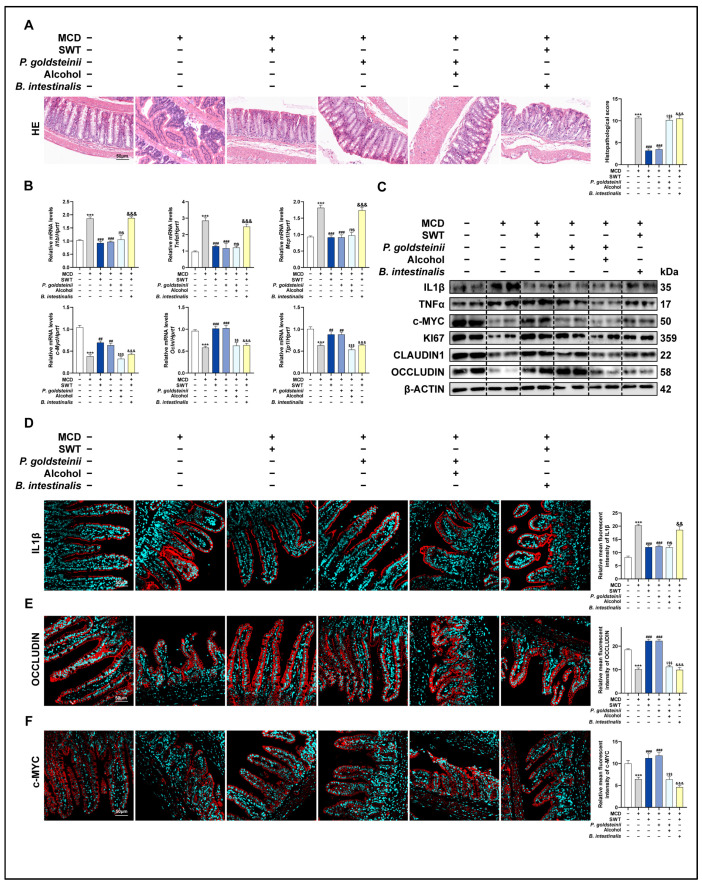
*P. goldsteinii* improved MCD diet-induced intestinal barrier dysfunction in mice. (**A**) H&E staining of intestine tissues (Scale bar, 50 μm). (**B**) Intestinal mRNA levels of *Tnfα*, *Il1b*, *Mcp1*, *c-Myc*, *Ocln*, and *Tjp1*. (**C**) Protein levels of OCCLUDIN, CLAUDIN-1, KI67, c-MYC, TNFα, and IL1β in intestine. (**D**–**F**) IF staining of (**D**) IL1β, (**E**) OCCLUDIN, and (**F**) c-MYC in intestine with quantification (Scale bar, 50 μm). Statistical significance: *** *p* < 0.001 vs. control group; ^##^ *p* < 0.01, ^###^ *p* < 0.001 vs. MCD group; ^$$^ *p* < 0.05, ^$$$^ *p* < 0.001 vs. MCD + *P. goldsteinii* group; ^&&^ *p* < 0.01, ^&&&^ *p* < 0.001 vs. MCD + SWT group; ns, no statistical differences vs. MCD + *P. goldsteinii* group. Data were presented as means ± SEM. One-way ANOVA with Tukey’s post hoc tests (*n* = 6).

To further delineate the intestinal effects of the two bacterial interventions and to determine whether these changes could influence the differential gut–liver axis phenotypes, we performed a systematic evaluation of ileal tissues across groups. Overall, supplementation with *P. goldsteinii* alone elicited a “repair-oriented” intestinal phenotype that was comparable to that observed with SWT. Histologically, the MCD group exhibited overt mucosal structural damage accompanied by inflammatory cell infiltration, whereas both the MCD + *P. goldsteinii* and MCD + SWT groups showed a marked improvement in villus/mucosal integrity and reduced inflammatory infiltration (Figure 7A). In contrast, when perturbing factors were introduced, this reparative trend was disrupted: no obvious barrier restoration was observed in either the MCD + *P. goldsteinii* + alcohol group or the MCD + SWT + *B. intestinalis* group (Figure 7A), suggesting that alcohol exposure or *B. intestinalis* may impede the establishment of intestinal benefits. With respect to inflammatory responses, *P. goldsteinii* likewise displayed an anti-inflammatory profile similar to SWT, which significantly blunted the MCD-induced transcriptional upregulation of IL1β, TNFα, and MCP1, and concomitantly reduced the TNFα and IL1β protein levels (Figure 7B,C). Furthermore, when examined from the perspective of barrier architecture and epithelial renewal, *P. goldsteinii* treatment increased the expression of tight junction-associated molecules (CLAUDIN-1, OCCLUDIN, and TJP1), as well as proliferation and renewal markers (c-MYC and KI67), with an overall magnitude comparable to that achieved by SWT (Figure 7B,C and Figure S9A). However, the improvements in both the inflammation axis and the barrier/proliferation axis were substantially attenuated when *P. goldsteinii* was administered under alcohol exposure, or when *B. intestinalis* was supplemented during SWT treatment, indicating that the intestinal benefits are susceptible to perturbation. Immunofluorescence analyses further supported these trends at the spatial level: group-wise changes in inflammatory signals (IL1β and TNFα) and barrier/proliferation-related signals (OCCLUDIN and c-MYC) were consistent with the qPCR and immunoblotting results (Figure 7D–F, Figures S9B–D and S10A,B). Taken together, these findings suggest that *P. goldsteinii* supplementation can recapitulate key SWT-associated intestinal phenotypes—namely, attenuation of inflammation and promotion of barrier repair and epithelial renewal—whereas alcohol exposure or the addition of *B. intestinalis* compromises these intestinal benefits, potentially undermining gut–liver axis-mediated protection. Moreover, the antagonistic influence of *B. intestinalis* on SWT efficacy is more likely related to ecological competition or microbiome remodeling. However, the precise mechanism warrants further functional investigation.

### 2.9. P. goldsteinii Is a Key Microbiota for Improving MASLD, and Its Exogenous Supplementation Can Improve MASLD

**Figure 8:**
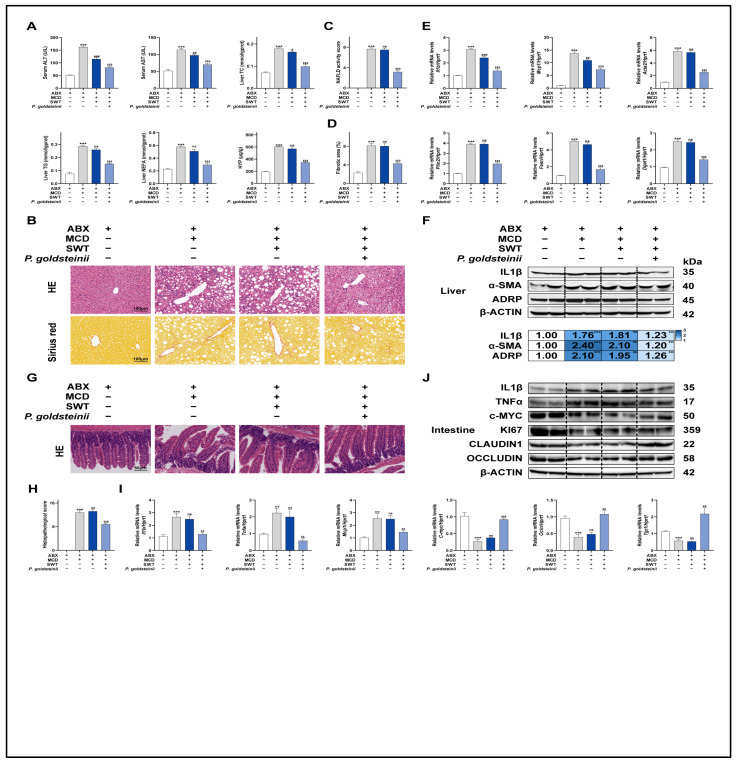
Supplementation of exogenous *P. goldsteinii* improved liver injury and intestinal barrier dysfunction in MASLD. (**A**) Serum ALT/AST and hepatic TC, TG, NEFA, HYP. (**B**) Liver H&E and Sirius red staining (Scale bar, 100 μm). (**C**) MASLD activity score. (**D**) Hepatic fibrosis score. (**E**) Hepatic mRNA levels of *Il1b*, *Mcp1*, *Acta2*, *Plin2*, *Fasn*, and *Dgat1*. (**F**) Protein levels of IL1β, α-SMA, and ADRP in the livers. (**G**) H&E staining of intestine tissues (Scale bar, 50 μm). (**H**) Intestinal histopathological score. (**I**) Intestinal mRNA levels of *Tnfα*, *Il1b*, *Mcp1*, *c-Myc*, *Ocln*, and *Tjp1*. (**J**) Protein levels of OCCLUDIN, CLAUDIN-1, KI67, c-MYC, TNFα, and IL1β in intestines. Statistical significance: ** *p* < 0.01, *** *p* < 0.001 vs. ABX group; ^#^ *p* < 0.1,^##^ *p* < 0.01, ^###^ *p* < 0.001 vs. ABX + MCD group; ^$$^ *p* < 0.05, ^$$$^ *p* < 0.001 vs. ABX + MCD + SWT group; ns, no statistical differences vs. ABX + MCD group.

To minimize the possibility that the complex indigenous microbiota could mask or exaggerate the effects of *P. goldsteinii*, we established an antibiotic-mediated microbiota-depletion (ABX) reduction system. After substantially diminishing the interference from resident microbes, we performed bacterial colonization and administration to evaluate the impact of *P. goldsteinii* on MASLD phenotypes and to assess the extent to which SWT efficacy depends on the gut microbial context. Under these conditions, compared with the ABX + MCD group, SWT showed only a downward trend in serum ALT and AST, whereas improvements in hepatic TG, NEFA, and HYP did not reach statistical significance. In contrast, supplementation with *P. goldsteinii* significantly improved all of the above biochemical indices (Figure 8A). These findings suggest that when the microbiota is markedly diminished, SWT confers a relatively limited amelioration of liver injury, whereas *P. goldsteinii* is more likely to exert a direct and broader protective capacity against multiple dimensions of hepatic damage. Histological evidence further supported this divergence. H&E and Sirius red staining revealed that, following ABX treatment, SWT produced only modest improvements in hepatic inflammatory infiltration and structural disruption, with a relatively limited impact on fibrosis-related alterations. By contrast, *P. goldsteinii* more robustly reduced inflammatory infiltration, promoted restoration of hepatic architecture, and decreased collagen deposition (Figure 8B–D). Consistently, immunofluorescence signals for IL1β, α-SMA, and ADRP exhibited the similar results (Figure S11A–C). At the level of mechanistic markers, qPCR and immunoblotting demonstrated that *P. goldsteinii* intervention concurrently downregulated inflammation-, fibrosis-, and lipid-accumulation-associated indices, whereas SWT under these conditions mainly manifested suppression of the inflammatory axis (e.g., Il1b and Mcp1), with less pronounced reductions in other injury-related markers (Figure 8E,F). The histopathological score of the intestine indicated that the related effect of SWT in repairing the mucosal barrier was markedly attenuated after ABX treatment. Intestinal H&E staining suggested that once the microbiota was substantially depleted, SWT no longer consistently displayed the phenotype of suppressing inflammation and preserving the villus architecture, whereas *P. goldsteinii* supplementation significantly alleviated inflammatory features and restored mucosal structural integrity (Figure 8G,H). At the molecular level, pro-inflammatory mediators (Il1b, Tnfa, and Mcp1) remained elevated in both the ABX + MCD and ABX + MCD + SWT groups, while barrier- and proliferation-related factors (c-Myc, Ocln, and Tjp1) remained reduced; notably, *P. goldsteinii* supplementation robustly reversed these alterations (Figure 8I,J). Immunofluorescence validation of IL1β, OCCLUDIN, and c-MYC was consistent with these findings (Figure S11D–F). Collectively, after ABX treatment cleared the intestinal flora, SWT exhibited a phenotype of limited protection for the liver and intestine. However, supplementation with *P. goldsteinii* not only improved intestinal inflammation and repaired the intestinal mucosal barrier but also had a restorative effect on liver damage. This indicates that *P. goldsteinii* may confer a more direct protective effect by maintaining mucosal integrity and regulating the gut–liver axis response.

## 3. Discussion

SWT, a traditional prescription, has been shown in our previous studies to ameliorate several liver diseases, including fibrosis, cholestasis and MASLD. In a CCl_4_-induced fibrosis model, SWT improved liver fibrosis by maintaining bile acid homeostasis along the gut–liver axis [21]. In cases of BDL-induced cholestasis, SWT alleviated hepatic inflammation and hepatocyte injury by suppressing neutrophil and macrophage polarization [19]. In the present study, SWT exerted hepatoprotective effects that were comparable to those of silymarin (positive control) in MCD diet-induced liver injury. Silymarin has been reported to support hepatocyte repair by promoting proliferation and limiting apoptosis, and to improve lipid metabolism by enhancing fatty-acid β-oxidation via AMPK activation and inhibiting ACC-mediated lipogenesis. Notably, our data suggest that alcohol exposure was associated with an attenuation of SWT efficacy in MCD-induced MASLD (Figure 1 and Figure 2). Acute high alcohol intake can elevate blood ethanol levels and aggravate steatosis and fibrosis [23,24,25]. Alcohol may also impair intestinal mucosal integrity and thereby reduce the absorption of orally administered bioactive constituents. In addition, because SWT is primarily metabolized in the liver, alcohol-induced hepatic dysfunction could plausibly alter SWT biotransformation and reduce its apparent efficacy. These possibilities are not mutually exclusive. Future work is needed to determine whether alcohol directly compromises key SWT constituents (e.g., by chemical interaction or altered pharmacokinetics) and/or whether alcohol-induced injury exceeds the therapeutic scope of SWT.

Bidirectional crosstalk between the liver and intestine is mediated by BAs, fatty acids, intestinal flora and other metabolites [26,27]. Beyond regulating lipid and immune homeostasis, BAs can shape the gut microbial community, whereas microbial enzymes deconjugate and transform primary bile acids into secondary BAs, thereby modulating bile-acid pools [28,29]. For example, *Lactobacillus* spp. can produce bile salt hydrolase, facilitating bile-salt biotransformation and potentially mitigating bile-acid-driven inflammation and cholestasis [30,31,32]. Consistent with this concept, we previously found that SWT increased circulating unconjugated BAs while decreasing conjugated BAs, which coincided with improved cholestatic liver injury via enterohepatic circulation [21]. Importantly, the MCD diet not only induces hepatic lipid accumulation but is also accompanied by progressive alterations in the intestinal microbiota and metabolites during hepatitis [33,34]. Therefore, we hypothesized that SWT benefits in MCD-induced MASLD may involve modulation of the intestinal microbiota and barrier function. Accumulating evidence links MASLD progression to gut dysbiosis and intestinal barrier function [35]. In our study, SWT was associated with improved intestinal barrier features, particularly in the small intestine, together with enhanced epithelial proliferation/differentiation (Figure 4 and Figure 5). Based on V3–V4 16S rRNA profiling and functional prediction, SWT increased several taxa at a resolution that was consistent with amplicon-based inference, including *Parabacteroides goldsteinii*-like taxa, *Bacteroides multiformis*-like taxa and *Bifidobacterium*-like taxa. These taxa showed positive associations with predicted functions related to the tight junction, the Wnt signaling pathway and fatty acid degradation. Therefore, we speculated that the role of SWT in repairing the intestinal mucosal barrier and resisting MASLD may be associated with the increased abundance of *P. goldsteinii*-like taxa. As noted by Li et al., *P. goldsteinii* can utilize the 7α-hydroxysteroid dehydrogenase to enhance 7-ketocholic acid production, activate Wnt signaling, and stimulate intestinal cell proliferation [36]. Fawad et al. found that *P. goldsteinii* was directly linked to the synthesis of the beneficial short-chain fatty acid, which helps to restore the intestinal mucosal barrier and prevent MASLD [37]. In our experiments, oral administration of *P. goldsteinii* improved hepatic and intestinal injury in MCD-fed mice, with effects that were comparable to SWT. Moreover, ABX treatment reduced SWT efficacy while lowering the abundance of putative beneficial taxa. As a result, we speculated that *P. goldsteinii* might be one of the effective intestinal bacterial species by which SWT improves MASLD. The TCM formula Si-Ni-San (SNS) is widely used for chronic liver disorders. In our recent work, SNS was associated with an increased abundance of *P. goldsteinii*, accompanied by higher levels of 7-ketose-cholic acid and SCFAs. These changes coincided with the restoration of the impaired intestinal mucosal barrier and amelioration of cholestatic liver injury [38]. Consequently, the active components of *Paeonia lactifora* Pall. (dry root) shared by SNS and SWT may regulate the proliferation of *P. goldsteinii*, potentially serving as an effective factor in the improvement of MASLD. This hypothesis warrants further investigation to identify the responsible compounds and their molecular targets. We found that SWT increased the abundance of *Bacteroides thetaiotaomicron*-like taxa, *Bifidobacterium pseudolongum*-like taxa, and *Klebsiella variicola*-like taxa. Li Hu et al. have demonstrated that *Bacteroides thetaiotaomicron* can reduce fat accumulation and protect against steatohepatitis, potentially by remodeling the intestinal microbial community and enhancing gut–liver folate and unsaturated fatty acid metabolism [39]. In addition, *Bifidobacterium pseudolongum* has been reported to alleviate inflammation and oxidative stress while modulating intestinal microbiota composition and hepatic metabolic pathways [40]. Therefore, we need to consider the potential of other probiotics, aside from *P. goldsteinii*, in combating MASLD. Because SWT has been reported to show no apparent toxicity at a moderate intake in humans [17], combining SWT with selected probiotics (e.g., *P. goldsteinii*-like taxa or *B. pseudolongum*-like taxa) may represent a promising strategy for MASLD patients with gut dysbiosis. In addition, given the intrinsic limitations of 16S rRNA amplicon sequencing, such as its inability to directly detect functional differences in microbiota, we will further investigate the pharmacological mechanisms linking the therapeutic effects of SWT to the modulation of the gut–liver axis within MASLD through metagenomic sequencing.

SWT was associated with reduced relative abundances of taxa annotated at the amplicon level, as potential pathobionts, including *Bacteroides intestinalis*-like taxa, and alcohol exposure were accompanied by a further reduction. Therefore, the relationship between alcohol and gut microbiota is not simply a straightforward positive or negative correlation; rather, it is likely influenced by underlying and complex regulatory mechanisms. This complexity undoubtedly presents challenges for research related to MASLD, which warrants further investigation. In parallel, alcohol has well-established detrimental effects on intestinal barrier integrity. It can disrupt epithelial cell membranes, contribute to villus injury, and downregulate tight-junction proteins, thereby impairing mucosal barrier function [41,42]. Given that alcohol not only causes damage to the liver and intestines but also has the potential to diminish the protective effects of medications and probiotics (Figure 7 and Figure 8), it is crucial to consider the impact of poor dietary habits on treatment efficacy when managing patients with MASLD.

## 4. Materials and Methods

### 4.1. Preparation of SWT

Commercial four herbs were used to prepare SWT: 17.5 g each of *Ligusticum chuanxiong* Hort., *Angelica sinensis* (Oliv.) Diels, *Paeonia lactiflora* Pall., and *Rehmannia glutinosa* Libosch. The herbs were fragmented into approximately 2–3 mm pieces, boiled and extracted in 560 mL distilled water for 1 h and then boiled in 560 mL for another 1 h. The mixture was filtered through gauze to obtain the filtrate, then evaporated under reduced pressure at 55 °C to 67.3 mL to obtain a concentration of 1.04 g crude drug/mL. Utilizing syringe filtration, SWT was filtered twice with 100 μm mesh and 0.45 μm filter, respectively. Finally, 65 mL SWT obtained through syringe filtration was stored at −20 °C.

### 4.2. Liquid Chromatograph Mass Spectrometer (LC-MS) Analysis of SWT

To perform identification of the chemical composition of SWT, liquid chromatograph mass spectrometer (LC-MS) analysis of the concentrated sample was conducted as described previously by [19]. Standard solutions of Turanose, Albiflorin, 3-*N*-butyl-4,5-dihydrophthalide, Senkyunolide I, Gentianose, 5-Hydroxymethylfurfural, Paeoniflorin sulfite, Manninotriose, Isoferulic acid, Benzoylalbiflorin, Isochlorogenic acid A, Camelliaside A, Rehmannioside D, Lactiflorin, Ligustilide, Levistolide A, Cnidilide, Oxypaeoniflorin, Hesperidin, Isomartynoside, Cordycepin, and Verbascoside were prepared in methanol at concentrations of 3.1643, 1.3621, 0.9835, 2.5824, 0.7002, 0.3627, 2.1743, 1.9312, 0.7830, 2.8220, 0.4929, 0.5913, 1.7231, 4.8240, 0.3828, 1.3453, 2.1485, 0.6883, 2.4426, 0.4782, 1.1127, and 1.3422 mg/mL, respectively.

SWT was profiled by HPLC, using a Welch Ultimate™ C18 column (Welch Materials Technology Co., Ltd., Shanghai, China) (250 mm × 4.6 mm i.d., 5 μm). The mobile phase consisted of 0.5% phosphomolybdate in water (solvent A) and acetonitrile (solvent B). Separation was achieved with the following gradient: 0–5 min, 5% B; 5–20 min, 5–15% B; 20–32 min, 15% B; 32–41 min, 15–40% B; 41–55 min, 40% B; 55–70 min, 40–80% B; 70–75 min, 80–95% B; and 75–80 min, 95% B. The flow rate was set at 1.0 mL/min, the injection volume was 10 μL, and chromatograms were monitored at 254 nm.

### 4.3. Animal Studies

C57BL/6J mice (7 weeks old, 18–20 g, male) were purchased from SIBEIFU Biotechnology Co, Ltd. (Beijing, China). Mice were bred under a temperature of 22 ± 2 °C and a humidity of 40 ± 10% conditions with a cycle of 12 h light/12 h dark and fed with an unlimited chow diet and sterile water. The MASLD model was induced by feeding mice a MCD diet based on the Ain-76 standard, which contained 10% fat and lacked methionine and choline supplementation, for a duration of 6 weeks. Additionally, mice stimulated by alcohol were provided with MCD feed for the same period and were administered 30% alcohol at a dosage of 10 µL/g three times a week. In the first experiment in Figure 1, Figure 2 and Figure 3, mice were randomly divided into twelve groups (*n* = 6), including (1) Control group, (2) MCD group, (3) Alcohol group, (4) MCD + SWT low dose (L) group, (5) MCD + SWT medium dose (M) group, (6) MCD + SWT high dose (H) group, (7) MCD + Silymarin group, (8) MCD + Alcohol group, (9) MCD + Alcohol + SWT low dose (L) group, (10) MCD + Alcohol + SWT medium dose (M) group, (11) MCD + Alcohol + SWT high dose (H) group, and (12) MCD + Alcohol + SWT + Silymarin group. Mice in Groups (4)–(6) and Groups (9)–(11) were given intragastrical administration of SWT (L) (2.6 g/kg), SWT (M) (5.2 g/kg) and SWT (H) (10.4 g/kg) daily for 5 weeks. Mice in Groups (7) and (12) were given intragastrical administration of 150 mg/kg silymarin daily for 5 weeks. In the second experiment in Figure 6 and Figure 7, mice were randomly divided into six groups (*n* = 6), including the (1) Control group, (2) MCD group, (3) MCD + SWT (5.2 g/kg) group, (4) MCD + *P. goldsteinii* group, (5) MCD + *P. goldsteinii* + Alcohol group and (6) MCD + SWT (5.2 g/kg) + *B. intestinalis* group. Mice in Groups (3) and (6) were gavaged daily with SWT at a medium dose (5.2 g/kg) for 5 weeks. Mice in Groups (4) and (5) received *P. goldsteinii* (2 × 10^8^ CFU) by oral gavage three times per week for 5 weeks. Mice in Group (6) received *B. intestinalis* (2 × 10^8^ CFU) by oral gavage three times per week for 5 weeks. In the third experiment in Figure 8, mice were assigned to four groups (*n* = 6): (1) antibiotic cocktail experiment (ABX) group, (2) ABX + MCD group, (3) ABX + MCD + SWT group, and (4) ABX + MCD + SWT + *P. goldsteinii*. Mice in Groups (2)–(4) were gavaged daily with 300 μL of ABX cocktail (ampicillin sodium, vancomycin hydrochloride, neomycin sulfate, and metronidazole prepared at 8:4:8:8 g/L and diluted in PBS) for 5 weeks. Mice in Groups (3) and (4) were given intragastrical administration of SWT (M) (5.2 g/kg) daily for 5 weeks. In addition, mice in Group (4) were gavaged with *P. goldsteinii* (2 × 10^8^ CFU) three times per week for 5 weeks. Animal experiments were carried out in accordance with the “Guide for the Care and Use of Laboratory Animals” and the “Principles for the Utilization and Care of Vertebrate Animals” guidelines, and all procedures were approved by The Institutional Animal Care and Use Committee at Beijing University of Chinese Medicine (BUCM-4-2020121003-4190, the Approval Date is December 2020, and BUCM-2023082201-3058, the Approval Date is August 2023).

### 4.4. Immunofluorescence Staining

A liver specimen was collected from the periportal region of a major hepatic lobe as an approximately 1 cm × 1 cm × 1 cm tissue block, and a 2 cm segment of distal small intestine from the corresponding anatomical site was harvested. Tissues were gently rinsed in phosphate-buffered saline to remove residual blood and immediately fixed in 4% paraformaldehyde at 4 °C for 24–48 h. After fixation, samples were trimmed, placed in embedding cassettes, dehydrated through graded ethanol (70%, 80%, 95%, and 100%), and cleared in xylene. Tissues were then infiltrated with paraffin at 60 °C with two to three changes and embedded in paraffin molds with appropriate orientation. After the blocks had fully solidified, serial sections were cut on a rotary microtome at 3–5 μm thickness. Sections were floated on a 40–45 °C water bath, mounted onto glass slides, and baked at 60 °C for 1–2 h to ensure adhesion.

Prior to staining, slides were warmed at 60 °C to melt paraffin, deparaffinized in xylene three times for 5 min each, and rehydrated through graded ethanol (100%, 95%, 80%, and 70%; 5 min each), followed by rinsing in distilled water for 5 min. Antigen retrieval was performed by heating sections in antigen retrieval buffer at boiling temperature for 40 min, followed by cooling to room temperature. Endogenous peroxidase activity was quenched by incubation with hydrogen peroxide solution for 30 min. Sections were then blocked for 60 min with blocking buffer consisting of 10% goat serum, 0.1% Triton X-100, and 1% bovine serum albumin in phosphate-buffered saline to minimize nonspecific binding, and incubated at 4 °C overnight with primary antibodies against IL1β (dilution, 1:200), α-SMA (dilution, 1:400), ADRP (dilution, 1:200), Occludin (dilution, 1:200), and c-Myc (dilution, 1:100). The sections were then stained with DAPI to see the nuclei and treated with a secondary antibody labeled with fluorescein to detect specific protein localization. The images were examined using an Olympus FV3000 confocal laser scanning microscope (Tokyo, Japan).

### 4.5. The 16S rRNA Gene Sequencing and Analysis

Bacterial genomic DNA was isolated from intestinal contents using the MagPure Soil DNA LQ Kit (Magen, Guangzhou, China). The V3–V4 hypervariable regions of the bacterial 16S rRNA gene was amplified via polymerase chain reaction (PCR) utilizing universal primers. The PCR products were purified using the Agencourt AMPure XP beads (Beckman Coulter Co., Brea, CA, USA) and quantitatively analyzed using the Qubit dsDNA assay kit (Life Technologies, Carlsbad, CA, USA). Subsequently, the sample concentrations were adjusted to a level that was suitable for sequencing. Sequencing was performed on the Illumina NovaSeq6000 (Illumina, San Diego, CA, USA) which included two paired-end sequencing cycles, each reading 250 bases. The paired-end sequencing reads were merged into single contiguous sequences using Cutadapt, with primer sequences trimmed off. Low-quality sequences were filtered, denoised, and merged, and chimeric reads were detected and removed from the paired-end reads using the default parameters in DADA2 and QIIME2. The Shannon diversity index and the Chao1 diversity index were used to compute differences in alpha diversity. QIIME software (version 2020.11) was used to calculate beta diversity, which was then displayed in principal coordinates analysis (PCoA) plots. Analysis of similarities was used to assess the statistical significance (ANOSIM). The corresponding abundance association between samples and bacterial communities at the family level was visualized by creating a collinearity diagram using Circos software (version 0.66). *p* values < 0.05 and linear discriminant analysis (LDA) scores ≥ 3 were used to identify microorganisms that were significantly differentially expressed. These typical bacteria were subjected to function analysis using annotations from the Kyoto Encyclopedia of Genes and Genomes (KEGG). Finally, the software output representative reads, ASV abundance tables, and species annotation were performed using the Silva and ITS databases as independent references in the QIIME2 (version QIIME2-202011).

### 4.6. The Culture of P. goldsteinii and B. intestinalis

*P. goldsteinii* (ATCC BAA-1180) and *B. intestinalis* (ATCC 8483) were obtained from the American Type Culture Collection (ATCC, Manassas, VA, USA), and were cultivated at 37 °C in a liquid thioglycolate medium (BD), along with anaerobic blood agar plates (Creative, Taiwan, China). The routine growth conditions included a mixed anaerobic gas atmosphere composed of 90% nitrogen, 5% hydrogen, and 5% carbon dioxide. These conditions were maintained within a Whitley DG250 anaerobic chamber (Don Whitley, Bingley, UK). The culture of *P. goldsteinii* was diluted to the concentration of 1 × 10^9^ CFU/mL. Final concentrations of 12 mg/mL SWT were administered. The absorbance values for it were measured at 24 h intervals.

### 4.7. Biofilm Formation Assay

Overnight cultures of *P. goldsteinii* were adjusted in liquid thioglycolate broth to an inoculum of OD_600_ = 0.1. Aliquots (96-well flat-bottom plate) were incubated at 37 °C on an orbital shaker (75 rpm) for 3 h to allow for biofilm formation. The spent medium was discarded and wells were rinsed three times with PBS to remove non-adherent cells. Subsequently, 500 µL of XTT working solution (KeyGEN Bio-Tech, Nanjing, China) was added to each well. The plate was incubated in the dark at 37 °C for 4 h, after which absorbance was read at 450 nm using a microplate spectrophotometer.

### 4.8. Statistical Analysis

The data were subjected to at least three replicates, with the mean ± standard error of the mean (SEM) being presented and analyzed using the one-way analysis of variance (ANOVA) method in GraphPad Prism 9.0. A *p* value of less than 0.05 was considered to be statistically significant.

Supplementary files containing additional method information and relevant specifics were made available online.

## 5. Conclusions

In summary, our research found that the MCD diet significantly altered the gut microbiota of mice and demonstrated that SWT might exert a therapeutic effect on MASLD by raising the abundance of potentially beneficial bacteria and reducing the abundance of pathogenic bacteria. Furthermore, alcohol-induced intestinal mucosal barrier damage could interfere with the therapeutic efficacy of SWT. This study offers new insights into the mechanisms by which SWT improves MASLD injury and provides a foundation for the development of *P. goldsteinii*-like taxa as a potential new therapeutic probiotic for treating MASLD.

## Data Availability

The original contributions presented in this study are included in the article/Supplementary Material. Further inquiries can be directed to the corresponding authors.

## Supplementary Materials

The following supporting information can be downloaded at: https://www.mdpi.com/article/10.3390/ph19030400/s1. Figure S1: Identification of active ingredients in SWT. Figure S2: SWT inhibited the inflammatory response, fibrosis and lipid accumulation caused by the MCD diet. Figure S3: Alcohol interfered with the hepatic protective role of SWT in MASLD. Figure S4: SWT improved the dysfunction of intestinal barrier in mice with MASLD, whereas alcohol exhibited the contrary effect. Figure S5: SWT inhibited intestinal inflammation in mice with MASLD, whereas alcohol exhibited the contrary effect. Figure S6: SWT and alcohol change the gut microbiota and their associated biological functions in MASLD mice. Figure S7: SWT favored the growth of *P. goldsteinii* in vitro. Figure S8: *P. goldsteinii* reduced the inflammatory response, fibrosis and lipid accumulation caused by the MCD diet, whereas *B. intestinalis* exhibited the contrary effect. Figure S9: *P. goldsteinii* reduced the inflammatory response in MASLD, whereas *B. intestinalis* exhibited the contrary effect. Figure S10: *P. goldsteinii* improved the dysfunction of intestinal barrier in MASLD, whereas *B. intestinalis* exhibited the contrary effect. Figure S11: *P. goldsteinii* improved the inflammatory response and the dysfunction of intestinal barrier in MASLD. Figure S12: The original picture of Western blot. Table S1: The drug materials and their authentication in SWT. Table S2: The concentration of main chemical compositions in SWT. Table S3: Primers used in qPCR (Mice). References [20,38] are cited in Supplementary Materials.

## Abbreviations

ABX	antibiotic cocktail experiment
ACTA2	actin alpha 2
ADRP	adipose differentiation-related protein
ALT	alanine aminotransferase
AST	aspartate aminotransferase
ASV	amplicon sequence variant
BAs	bile acids
BDL	bile duct ligation
c-MYC	myelocytomatosis viral oncogene homolog
DGAT1	diacylglycerol acyltransferase 1
FASN	fatty acid synthase
H&E	hematoxylin and eosin
HYP	hydroxyproline
IBD	inflammatory bowel disease
IFNα	interferon alpha
IL1β	interleukin 1 beta
LDA	linear discriminant analysis
MASLD	metabolic dysfunction-associated steatotic liver disease
MCD	methionine–choline deficiency
MCP1	monocyte chemoattractant protein 1
NEFA	non-esterified fatty acids
OCLN	occludin
PLIN2	perilipin 2
SWT	Si-Wu-Tang
TC	total cholesterol
TJP1	tight junction protein 1
TG	triglyceride
TNFα	tumor necrosis factor alpha
α-SMA	α-smooth muscle actin
