# Supplementary material for: Si-Wu-Tang Targets Microbiota Homeostasis and Intestinal Mucosal Barriers to Provide Protection Against MASLD by Favoring P. goldsteinii-like Taxa Colonization
Source: Pharmaceuticals (Basel). 2026 Feb 28;19(3):400. doi: 10.3390/ph19030400 (PMC13029096; doi:10.3390/ph19030400)
Supplement: Supplementary file 1 [file pharmaceuticals-19-00400-s001.zip › pharmaceuticals-4094000-supplementary.pdf]

1. Supplementary materials and methods

1.1 Materials

The four herbs of SWT, including *Ligusticum chuanxiong* Hort. (Dry rhizome), *Paeonia lactifora* Pall. (Dry root), *Angelica sinensis* (Oliv.) Diels (Dry root) and *Rehmannia glutinosa* Libosch. (Processed products of dry root tuber) were all purchased from Beijing Tongrentang (Group) Co., Ltd. (China) and identifed by Prof. Liu from the School of Chinese Materia Medica, Beijing University of Chinese Medicine. Antibody against IL1β (16806-1-AP), β-ACTIN (66009-1-lg), ADRP (15294-1-AP), Occludin (27260-1-AP) ,and KI67 (27309-1-AP) were purchased from Proteintech (Rosemont, USA). Antibodies against alpha-smooth muscle actin (α-SMA) (19245S) and c-Myc (18583S) were obtained from Cell Signaling Technology (Danvers, USA). Antibodies against Claudin-1 (sc-166338) and TNFα (sc-52746) were obtained from Santa Cruz Biotechnology (Dallas, USA). The assay kits of Alanine aminotransferase (C009-2-1, including alanine aminotransferase matrix solution (CAS: 9000-86-6), 2,4-dinitrophenylhydrazine solution (CAS: 119-26-6), 4mol/L sodium hydroxide solution (CAS: 1310-73-2), 2 μmol/mL sodium pyruvate standard solution (CAS: 113-24-6), and 0.1mol/L phosphate buffer solution (CAS: 12111-21-6)), aminotransferase (C010-2-1, including AST matrix solution (CAS: 9000-97-9), 2,4-dinitrophenylhydrazine solution (CAS: 119-26-6), 4mol/L sodium hydroxide solution (CAS: 1310-73-2), 2 μmol/mL sodium pyruvate standard solution (CAS: 113-24-6), and 0.1mol/L phosphate buffer solution (CAS: 12111-21-6)), total cholesterol (A111-1-1, including alanine aminotransferase matrix solution (CAS: 9000-86-6), 2,4-dinitrophenylhydrazine solution (CAS: 119-26-6), 4mol/L sodium hydroxide solution (CAS: 1310-73-2), 2 μmol/mL sodium pyruvate standard solution (CAS: 113-24-6), and 0.1mol/L phosphate buffer solution (CAS: 12111-21-6)), triglycerides (A110-1-1, including 100mmol/L Tris HCL buffer (CAS: 1185-53-1), lipase (CAS: 9001-62-1), 0.5mmol/L ATP (CAS: 56-65-5), glycerol kinase (CAS: 9030-66-4), 3-phosphoglycerol oxidase (CAS: 9046-28-0), peroxidase (CAS: 9003-99-0), 1.4mmol/L 4-aminoantipyrine (CAS: 83-07-8), and 3mmol/L p-chlorophenol (CAS: 106-48-9)), non-esterified fatty acids (NEFA), and hydroxyproline (Hyp) were purchased from Nanjing Jiancheng Bioengineering Research Institute Co., Ltd (Nanjing, China). BCA protein quantitative detection kit (BN27109, including BCA Solution (CAS: 979-88-4) and

Cu^{2+} Solution (CAS: 7440-50-8)), ultra sensitive chemiluminescence detection kit (BN27109) and mouse lipopolysaccharide (LPS) elisa kit (BN53421) were purchased from Beijing Bairuiji Biotechnology Co., Ltd (Beijing, China). RNA isolater Total RNA Extraction Reagent, HiScript III RT SuperMix cDNA reverse transcription kit and AceQTM Universal SYBR qPCR Master Mix were purchased from Nanjing Vazyme Biotech Co., Ltd (Nanjing, China). Glycine (CAS: 517-28-2), Sodium chloride (CAS: 7647-14-5), Sodium phosphate,dibasic,anhydrous (CAS: 7558-79-4), Tris (CAS:77-86-1), and SDS (CAS:151-21-3) were purchased from Sangon Biotech (Shanghai) Co., Ltd. (Shanghai, China).

1.2 Western blotting

Tissues from the same anatomical sites of the major hepatic lobe and small intestine were collected from each mouse, homogenized, and lysed in RIPA buffer supplemented with a protease inhibitor cocktail. Lysates were centrifuged at 12,000 × g, and the supernatants were collected as total protein extracts. Protein concentration was determined using a BCA assay. Briefly, protein samples were added to a 96-well plate, mixed with BCA working reagent, and incubated at 37°C for 30 min in the dark. Absorbance was then measured at 562 nm using a microplate reader. A standard curve was generated using bovine serum albumin standards, and protein concentrations were calculated accordingly. After quantification, samples were mixed with loading buffer at a 1:4 ratio and denatured at 100°C for 15 min. Proteins were separated by SDS-PAGE and transferred onto PVDF membranes. Membranes were blocked with 5% non-fat milk at room temperature, followed by incubation with primary antibodies and then HRP-conjugated secondary antibodies. Signals were visualized using enhanced chemiluminescence and imaged. Band intensities were quantified using Quantity One software, normalized to β-ACTIN, and expressed as relative protein levels compared with the control group.

1.3 Biochemical assays

After sacrificed, mouse serum was centrifuged at 6000 g and collected for further experiments. The serum level of Alanine aminotransferase (ALT), aminotransferase (AST), total cholesterol (TC), triglycerides (TG), non-esterified fatty acids (NEFA), hydroxyproline (Hyp) and lipopolysaccharide (LPS) were measured by assay kits and

were analyzed according to the instructions.

1.4 Histopathology analysis

After fixing with 4% PFA and embedding in paraffin, the liver tissue and intestine. was sliced into 4.5 μm sections. The sections were dewaxed using xylene and then rehydrated with a series of graded alcohol solutions. Later, the slices were further staining with hematoxylin and eosin (HE) and Sirius red respectively. The histopathological scores were statistically analyzed using a blind method. The scoring criteria for the liver is the NAS scoring system: (1) steatosis (0-3 score), (2) ballooning degeneration (0-2 score), (3) inflammation (0-3 score). According to previous studies [38], the scoring criteria for the intestine tissues include the following indicators: (1) the extent of mucosal damage (0-3 score), (2) immune cells infiltration (0-3 score), (3) goblet cells deficiency (0-3 score), (4) thickening of the muscularis mucosa (0-3 score). The histopathological score was the summarization of these indicators.

1.5 Extracting RNA and qPCR

For each mouse, tissues were collected from the same anatomical sites of a major hepatic lobe and the small intestine. After gently rinsing the samples with PBS to remove surface blood, 700 μL TRIzol reagent was added to each tube and the tissues were thoroughly homogenized using a tissue homogenizer. Chloroform (200 μL) was then added to each tube to establish a phenol–chloroform phase-separation system, enabling the separation of RNA, DNA, and proteins based on differences in polarity; RNA was enriched in the upper aqueous phase, thereby improving extraction purity. Samples were vortexed vigorously, incubated at room temperature for 10 min to allow complete phase separation, and centrifuged at 12,000 × g for 15 min at 4°C. The upper clear aqueous phase containing RNA was carefully transferred to a new microcentrifuge tube. An equal volume of isopropanol was added and mixed gently by inversion to precipitate RNA. After centrifugation at 12,000 × g for 10 min at 4°C, the supernatant was discarded and a white RNA pellet was visible at the bottom of the tube. The pellet was washed with pre-chilled 75% ethanol prepared with PCR-grade water by gently inverting/rinsing to remove residual organic reagents. Samples were centrifuged at 7,500 × g for 5 min at 4°C, the ethanol was completely removed, and the

pellet was air-dried at room temperature until it became semitransparent. The RNA pellet was dissolved in an appropriate volume of PCR-grade water pre-warmed to 45°C. RNA concentration and purity were determined and the A260/A280 and A260/A230 ratios were recorded. RNA was then reverse-transcribed into cDNA using the HiScript III RT SuperMix kit. Quantitative real-time PCR was performed using AceQ™ Universal SYBR qPCR Master Mix. The mRNA levels of *Il1b, Acta2, Plin2, Fasn, Dgat1, Tnfα, Mcp1, c-Myc, Ocln, and Tjp1* were normalized to the internal reference *Hprt1*.

.

2. Supplementary figures

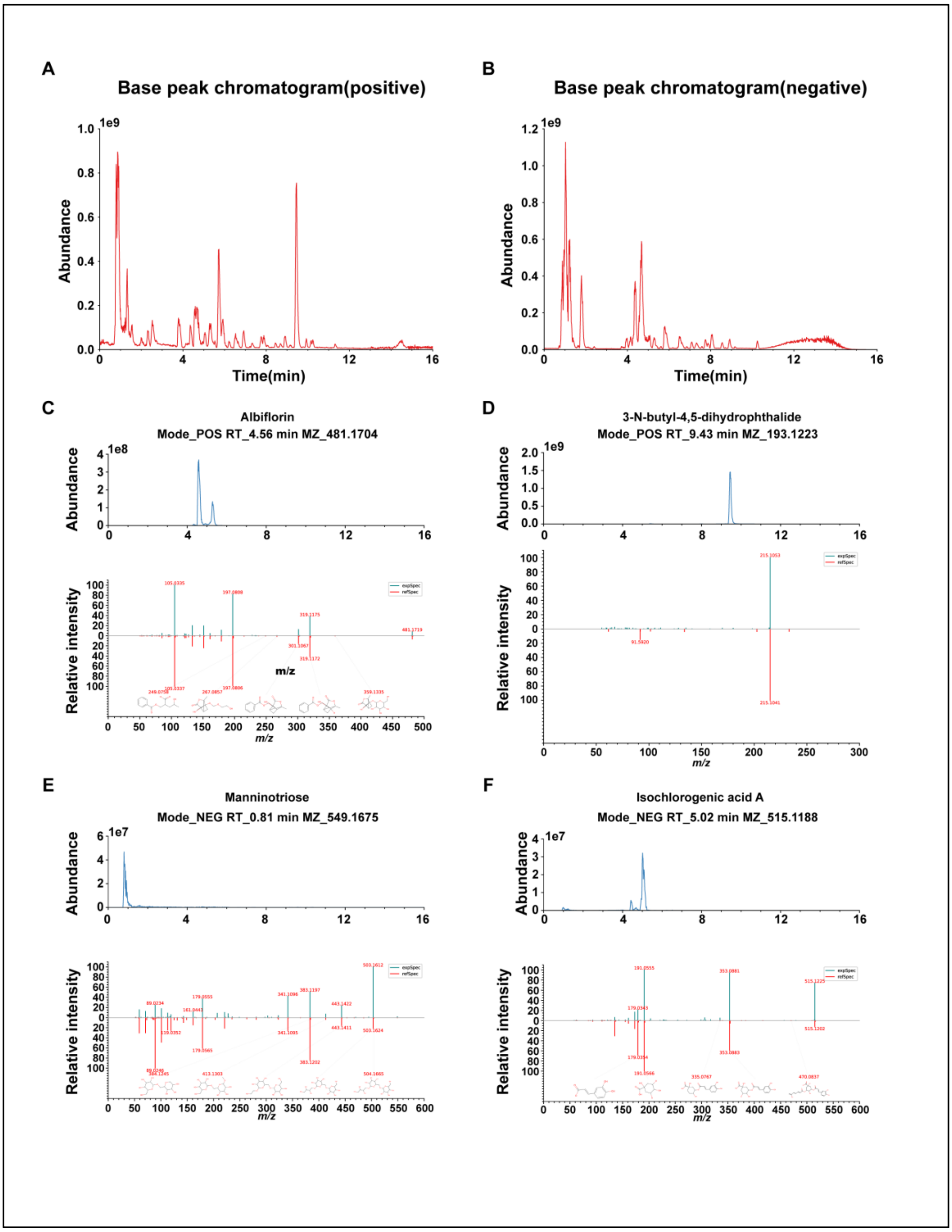

Figure S1. Identification of active ingredients in SWT. The total ion current chromatograms (TICCs) of the prepared SWT are shown in **(A)** positive and **(B)** negative ionization modes. Production ion mass spectra of **(C)** albiflorin, **(D)** 3-N-butyl-4,5-dihydrophthalide, **(E)** manninotriose, and **(F)** isochlorogenic acid A. The TICCs, exact mass information on precursors and fragment ions for the identified

compounds were all derived from our previous study [20].

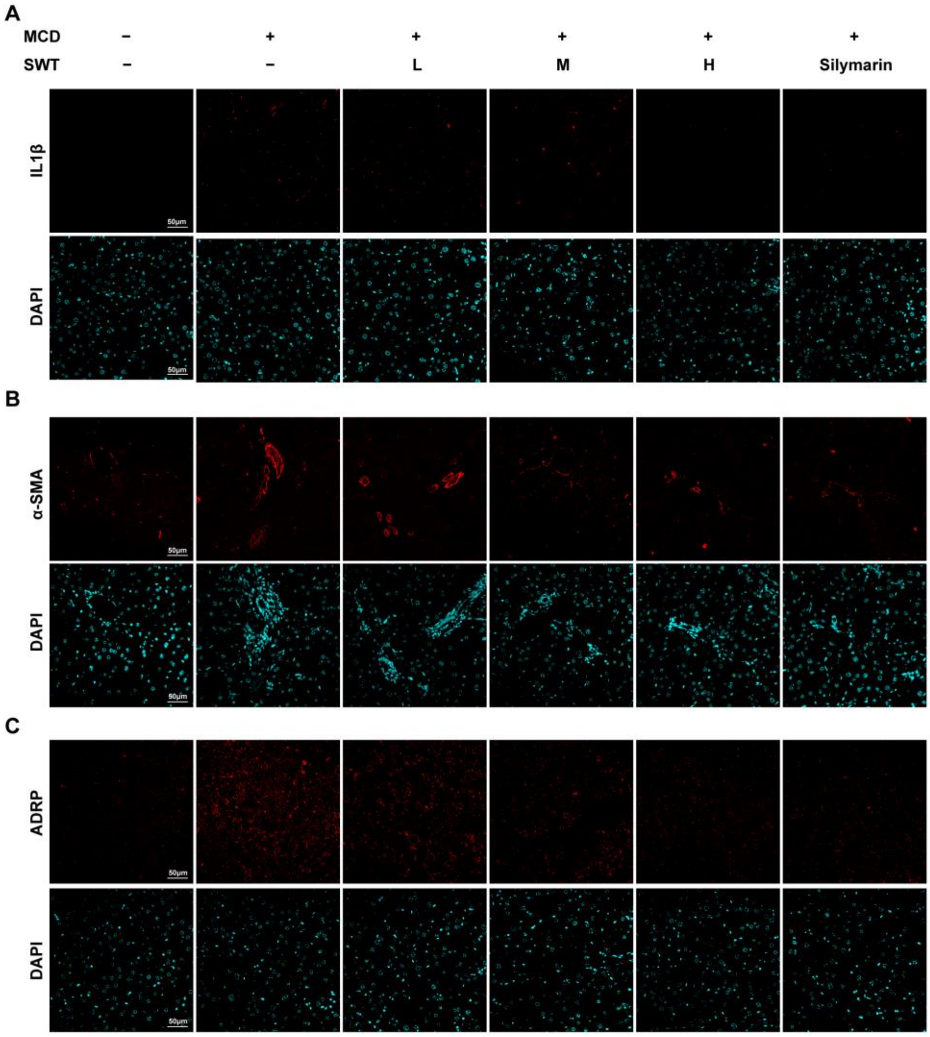

Figure S2. SWT inhibited the inflammatory response, fibrosis and lipid accumulation caused by the MCD diet**. (A-C)** IF staining of IL1β **(A)**, α-SMA **(B)**, and ADRP **(C)** in liver sections (scale bar, 50 μm).Nuclear staining by DAPI. Statistical significance: **P*<0.05, ***P*<0.01, ****P*<0.001 vs control group; #*P*<0.05, ##*P*<0.01 vs MCD group. Data were presented as means ± SEM. One-way ANOVA with Tukey's post-hoc tests (n = 6).

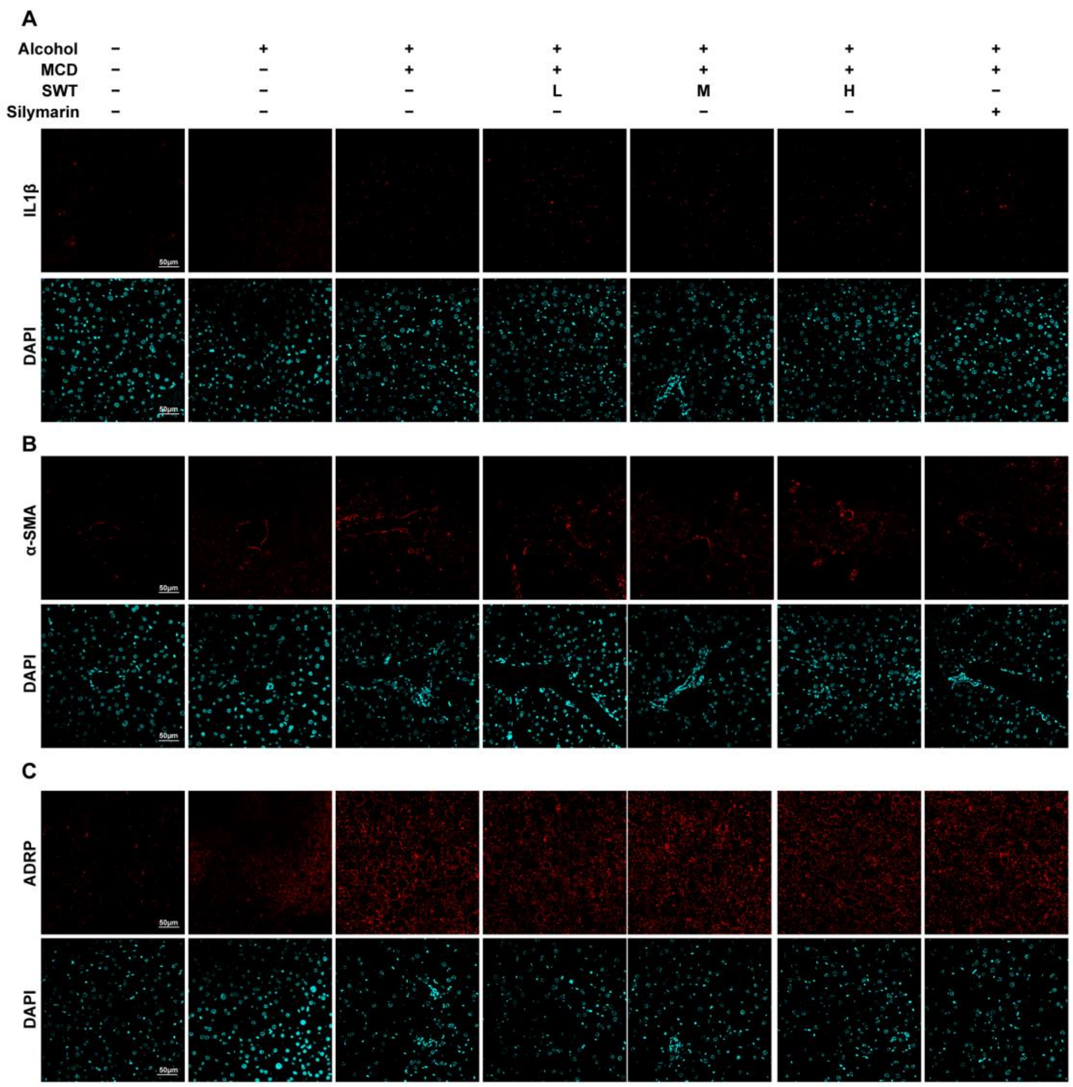

Figure S3. Alcohol interfered with the hepatic protective role of SWT in MASLD. **(A-C)** IF staining of IL1β **(A)**, α-SMA **(B)**, and ADRP **(C)** in liver sections (scale bar, 50 μm) Nuclear staining by DAPI. Statistical significance: ***P*<0.01, ****P*<0.001vs control group; No statistical differences among MCD + alcohol, MCD + alcohol + SWT low dose (L), MCD + alcohol + SWT medium dose (M), MCD + alcohol + SWT high dose (H), and MCD + alcohol + silymarin group. Data were presented as means ± SEM. One-way

ANOVA with Tukey’s post-hoc tests (n = 6).

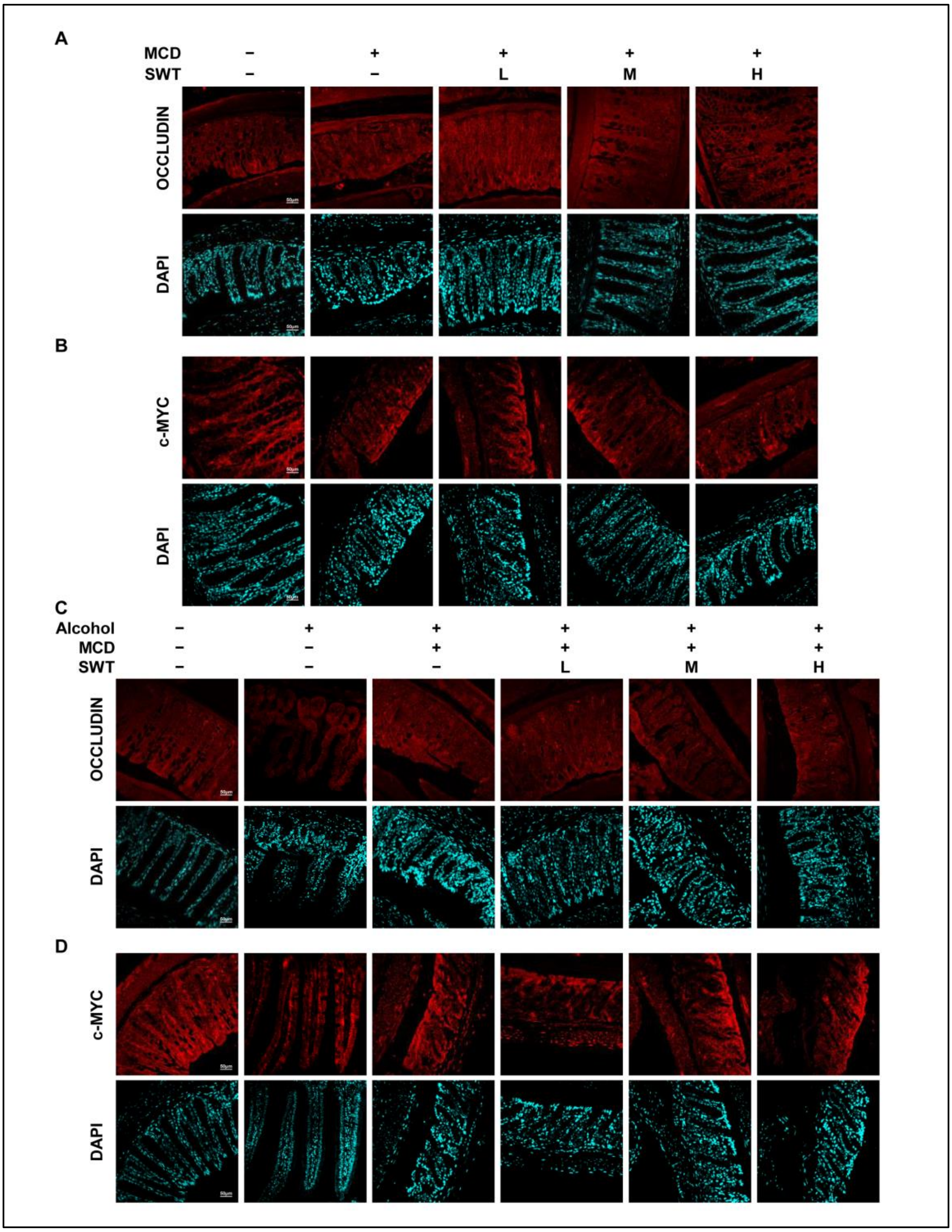

Figure S4. SWT improved the dysfunction of intestinal barrier in mice with MASLD, whereas alcohol exhibited the contrary effect. **(A-D)** IF staining of **(A, C)** OCCLUDIN and **(B, D)** c-MYC in intestine tissues (scale bar, 50 μm). Nuclear staining by DAPI.

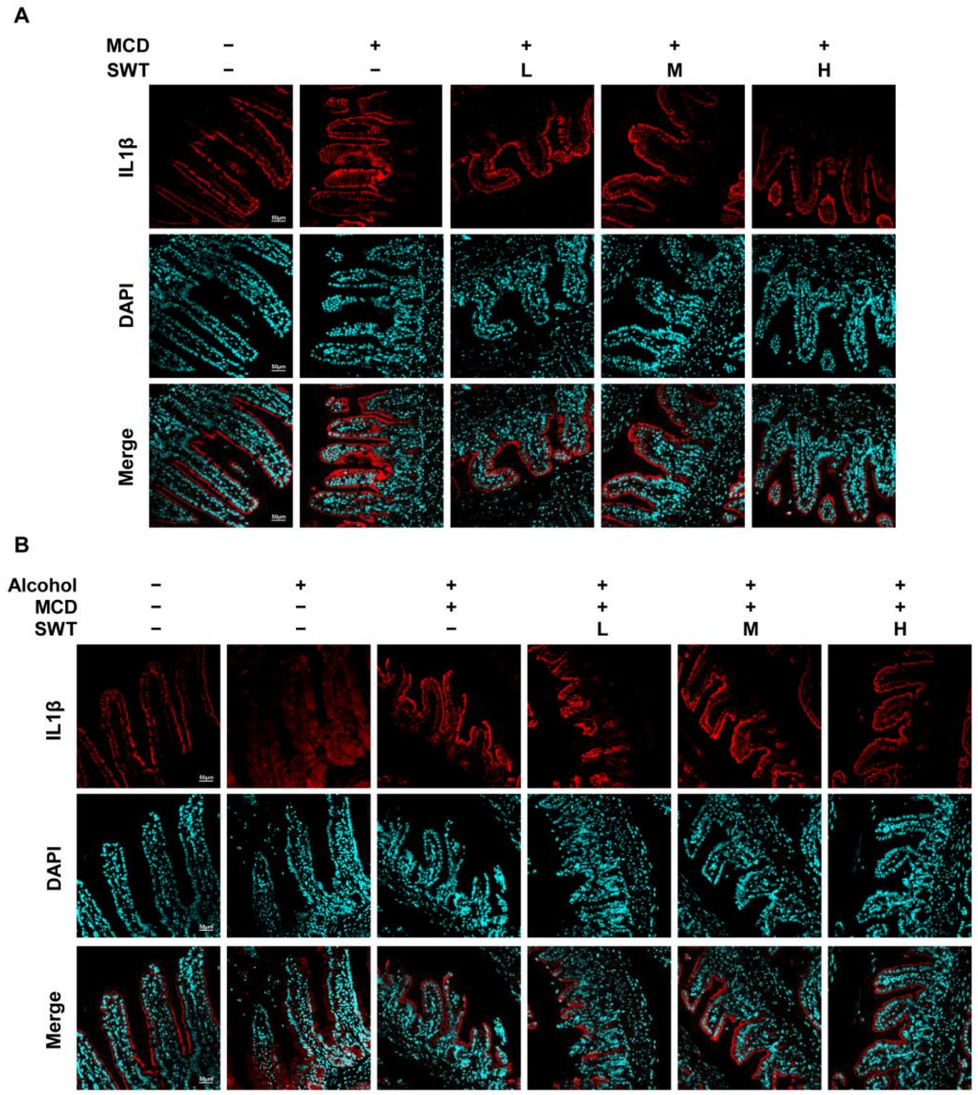

Figure S5. SWT inhibited intestinal inflammation in mice with MASLD, whereas alcohol exhibited the contrary effect. **(A, B)** IF staining of IL1β in intestine tissues (scale bar, 50 μm).

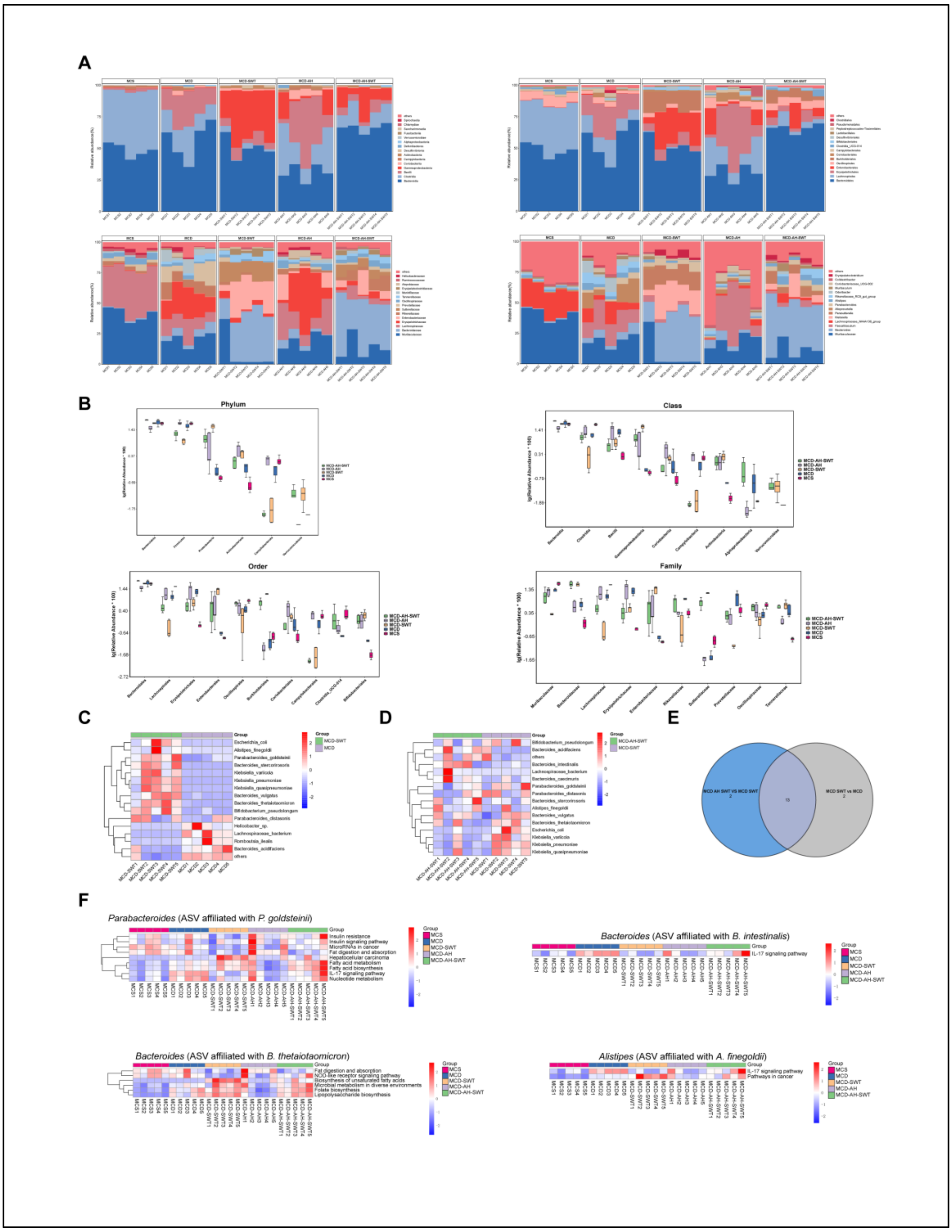

Figure S6. SWT and alcohol change the gut microbiota and their associated biological functions in MASLD mice. **(A)** Taxonomic composition at the class, order, family, and genus levels. **(B)** Top 10 taxa across the phylum, class, order, and family levels. **(C-D)** Predicted KEGG pathway profiles across groups. **(E)** Venn diagram of differential species between MCD+AH+SWT vs MCD+SWT and MCD+SWT vs MCD. **(F)** Predicted KEGG pathways associated with differential species

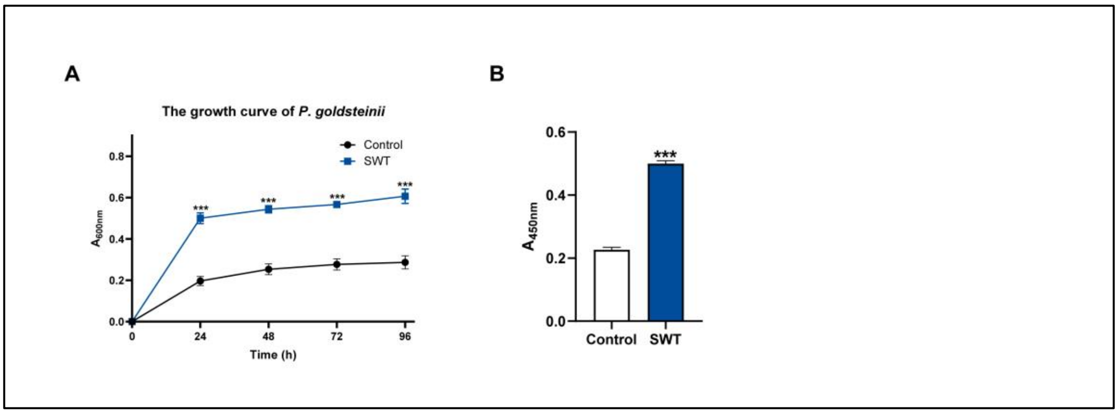

Figure S7. SWT favored the growth of *P. goldsteinii in vitro*. **(A)** The growth curve of *P. goldsteinii*. **(B)** The A_{450nm} values in the biofilm assay.

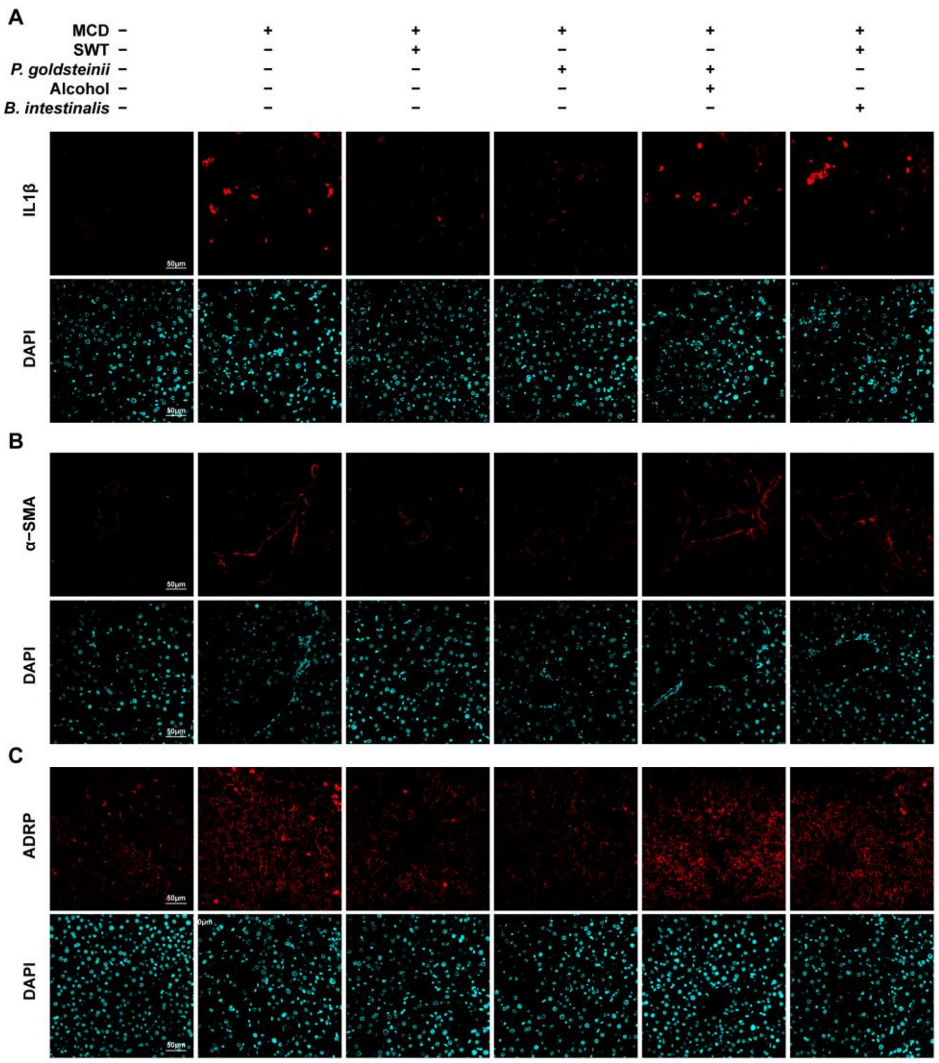

Figure S8. *P. goldsteinii* reduced the inflammatory response, fibrosis and lipid accumulation caused by the MCD diet, whereas *B. intestinalis* exhibited the contrary effect. **(A-C)**. IF staining of IL1β **(A)**, α-SMA **(B)**, and ADRP **(C)** in liver sections (scale bar, 50 μm)Nuclear staining by DAPI.

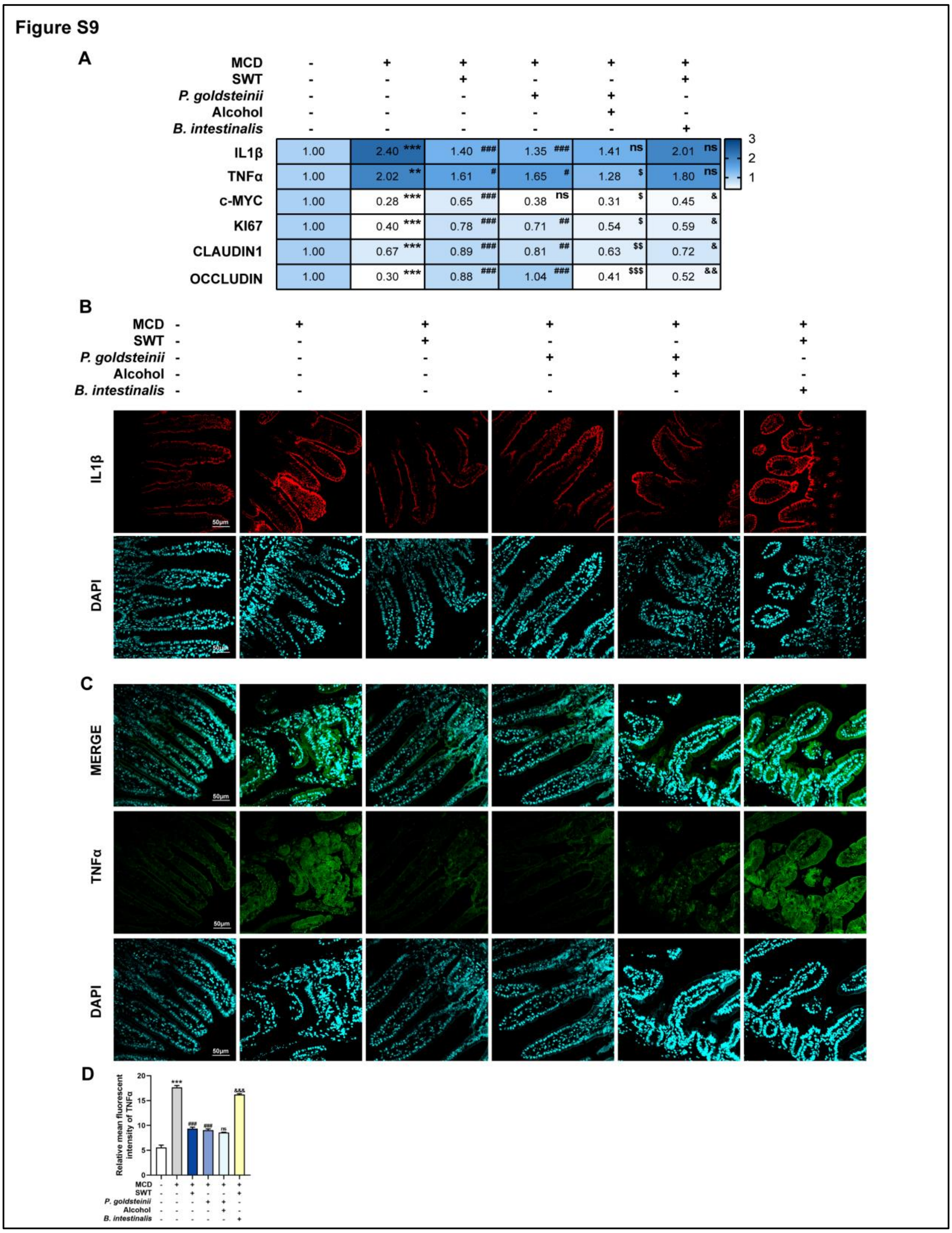

Figure S9. *P. goldsteinii* reduced the inflammatory response in MASLD, whereas *B. intestinalis* exhibited the contrary effect. **(A)** Protein levels of IL1β, TNFα, c-MYC, KI67, CLAUDIN-1, and OCCLUDIN in intestines. **(B-C)** IF staining of **(B)** IL1β and **(C)** TNFα in the intestines (scale bar, 50 μm). Nuclear staining by DAPI. **(D)** Relative mean fluorescent intensity of TNFα in the intestines.Data were presented as means ± SEM. Statistical significance: $^{**}P<0.01$, $^{***}P<0.001$ vs control group; $^{\#}P<0.05$, $^{\#\#}P<0.01$,

$^{###}P$<0.001 vs MCD group; $^{\$}P$<0.05, $^{\$\$}P$<0.01, $^{\$\$\$}P$<0.001 vs MCD + *P. goldsteinii* group; $^{\&}P$<0.05, $^{\&\&}P$<0.01, $^{\&\&\&}P$<0.001 vs MCD + SWT group. One-way ANOVA with Tukey's post-hoc tests (n = 6).

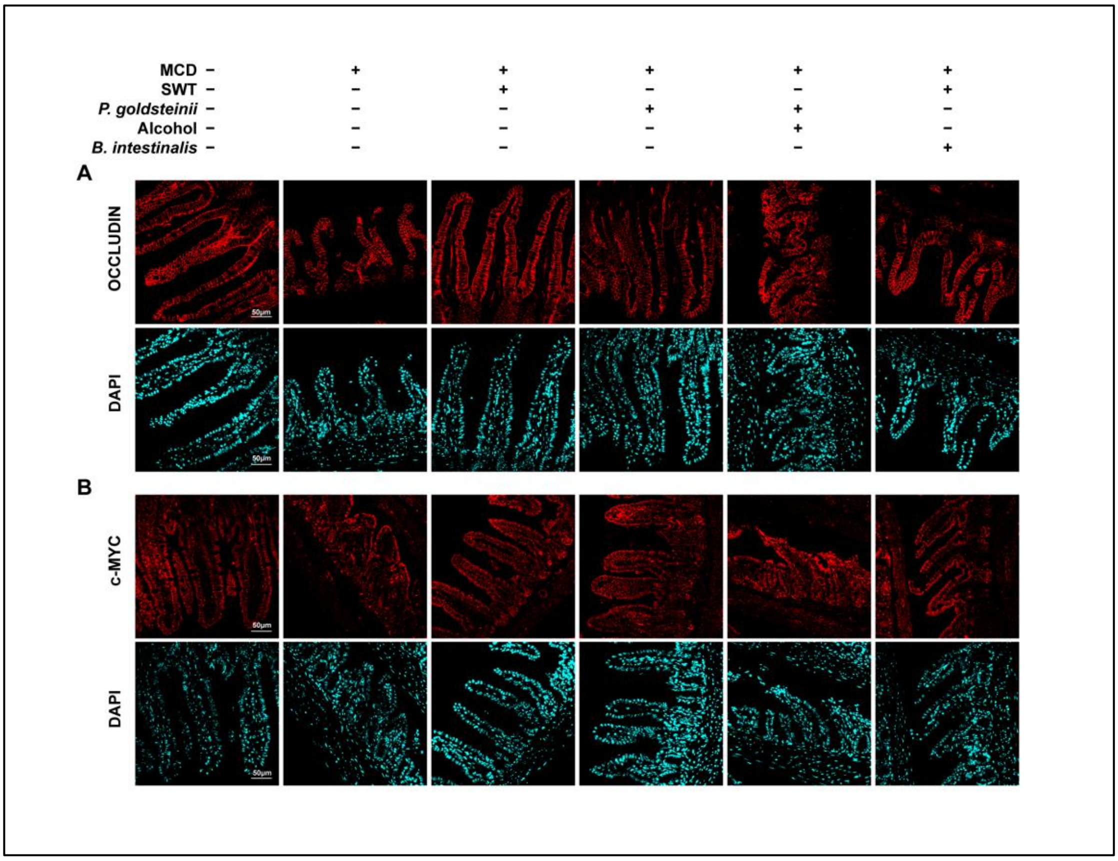

Figure S10. *P. goldsteinii* improved the dysfunction of intestinal barrier in MASLD, whereas *B. intestinalis* exhibited the contrary effect. Staining of **(A)** OCCLUDIN and **(B)** c-MYC in intestines (scale bar, 50 μm). Nuclear staining by DAPI.

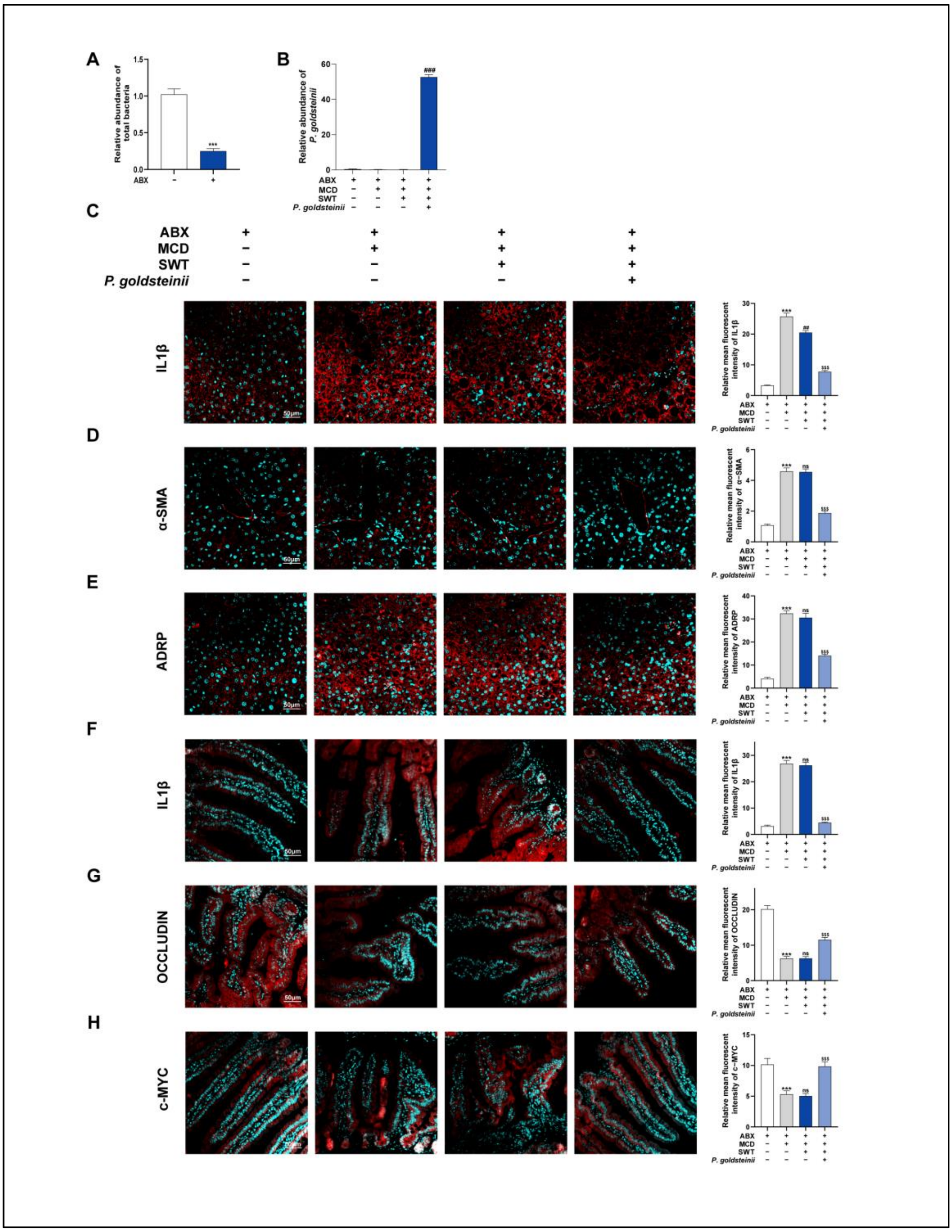

Figure S11. *P. goldsteinii* improved the inflammatory response and the dysfunction of intestinal barrier in MASLD. **(A-B)** Relative abundance of **(A)** total bacteria and **(B)** *P. goldsteinii* in feces. **(C-E)**. IF staining of**(C)** IL1β, **(D)** *α*-SMA and **(E)** ADRP in livers (scale bar, 50 μm). **(F-H)** IF staining of **(F)** IL1β, **(G)** OCCLUDIN and **(H)** c-MYC in intestines (scale bar, 50 μm). Nuclear staining by DAPI.

Figure 1.(G) Protein levels of IL1β, α-SMA, ADRP, and β-ACTIN in the livers.

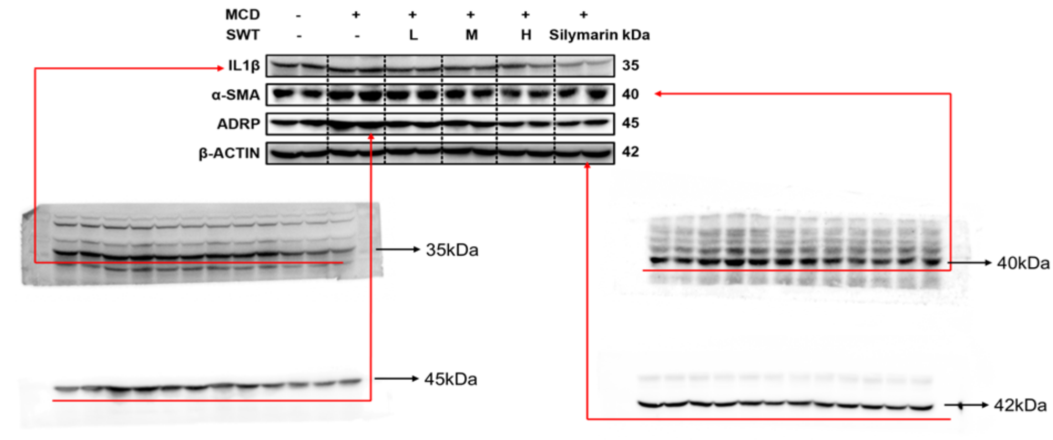

Figure 2.(G) Protein levels of IL1β, α-SMA, ADRP, and β-ACTIN in the livers.

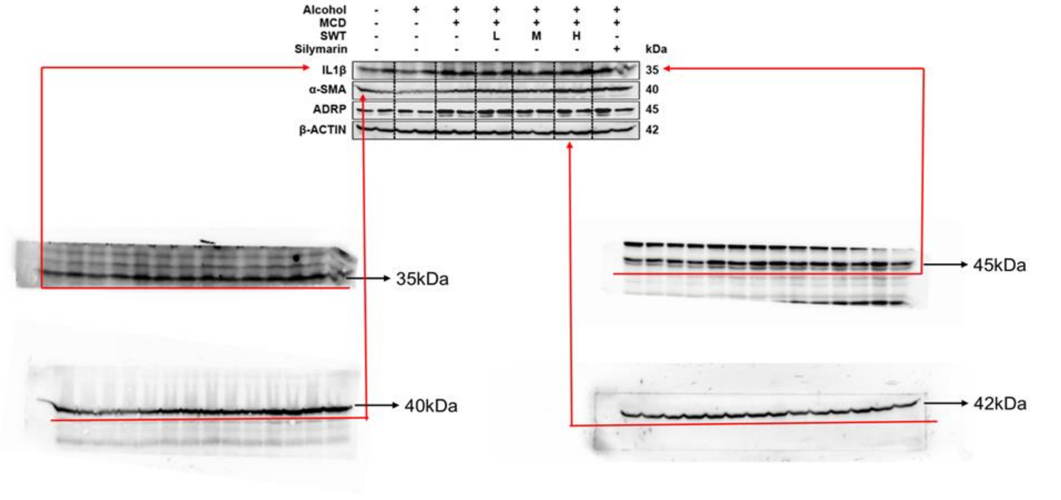

Figure 3.(C) Protein levels of OCCLUDIN, CLAUDIN-1, KI67, c-MYC, TNFα, IL1β, and β-ACTIN in intestines.

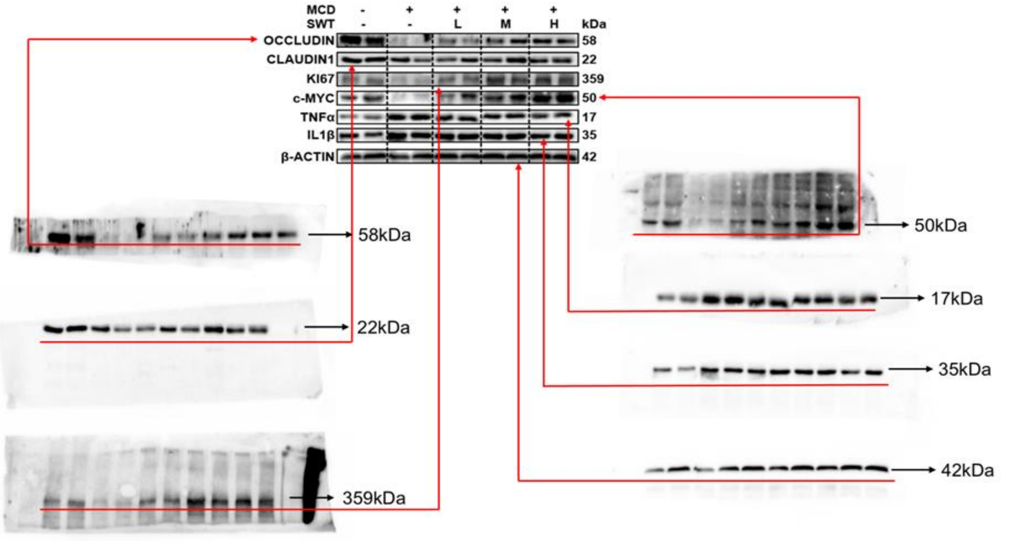

Figure 3.(H) Protein levels of OCCLUDIN, CLAUDIN-1, KI67, c-MYC, TNFα, IL1β, and β-ACTIN in intestines.

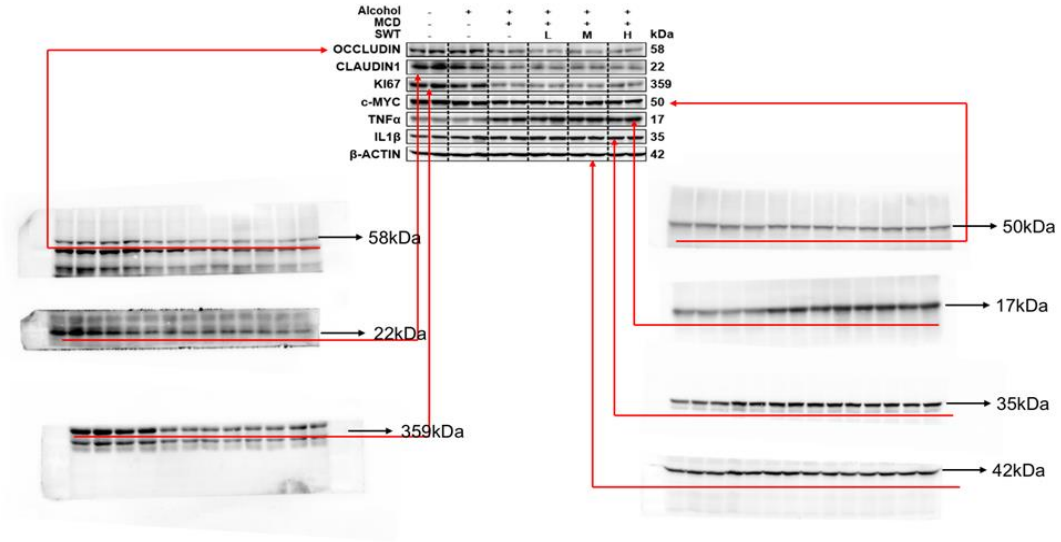

Figure 6.(D) Protein levels of IL1β, α-SMA, ADRP, and β-ACTIN in the livers.

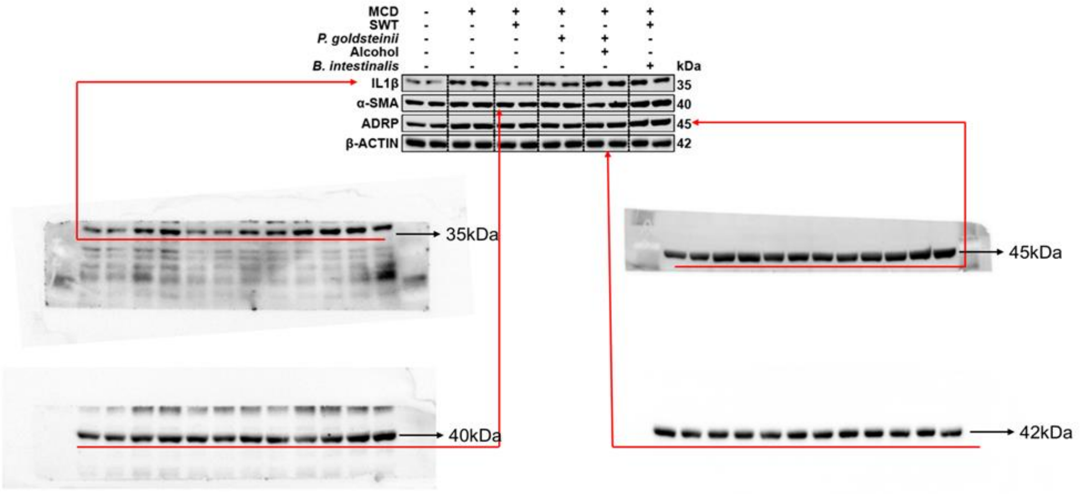

Figure 7.(C) Protein levels of OCCLUDIN, CLAUDIN-1, KI67, c-MYC, TNFα, IL1β, and β-ACTIN in intestines.

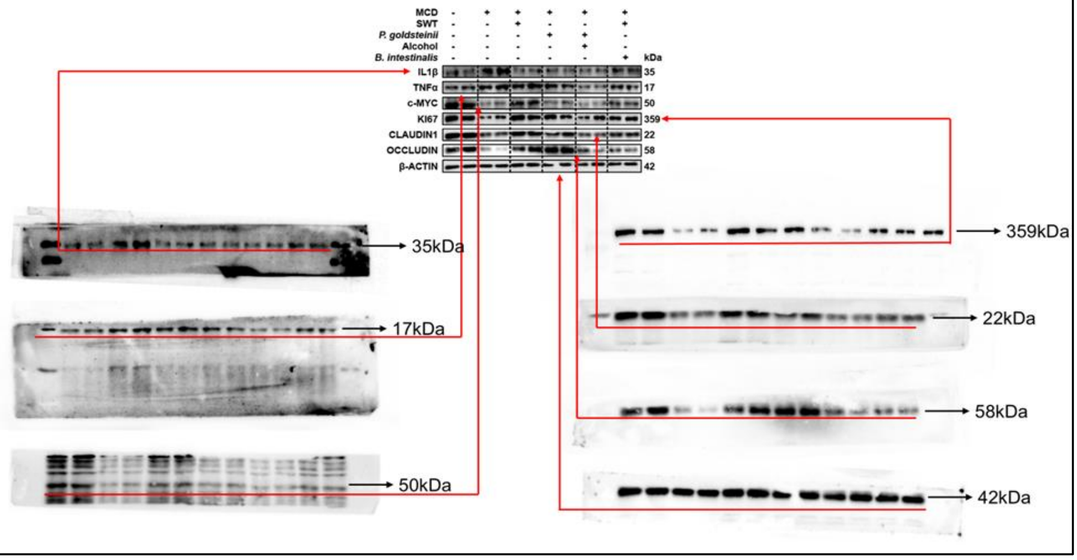

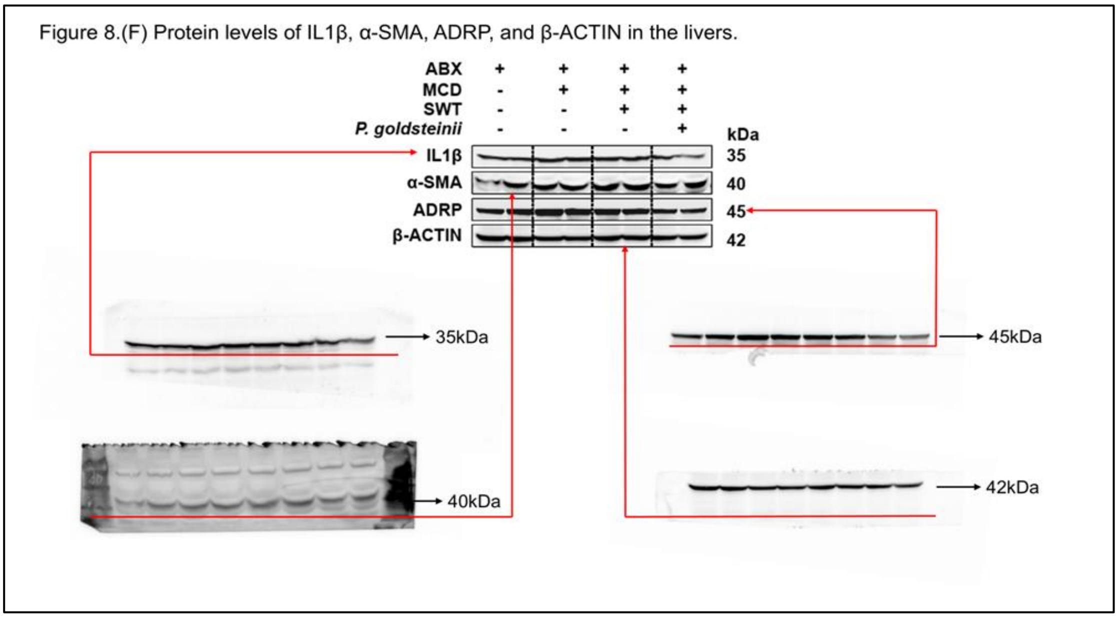

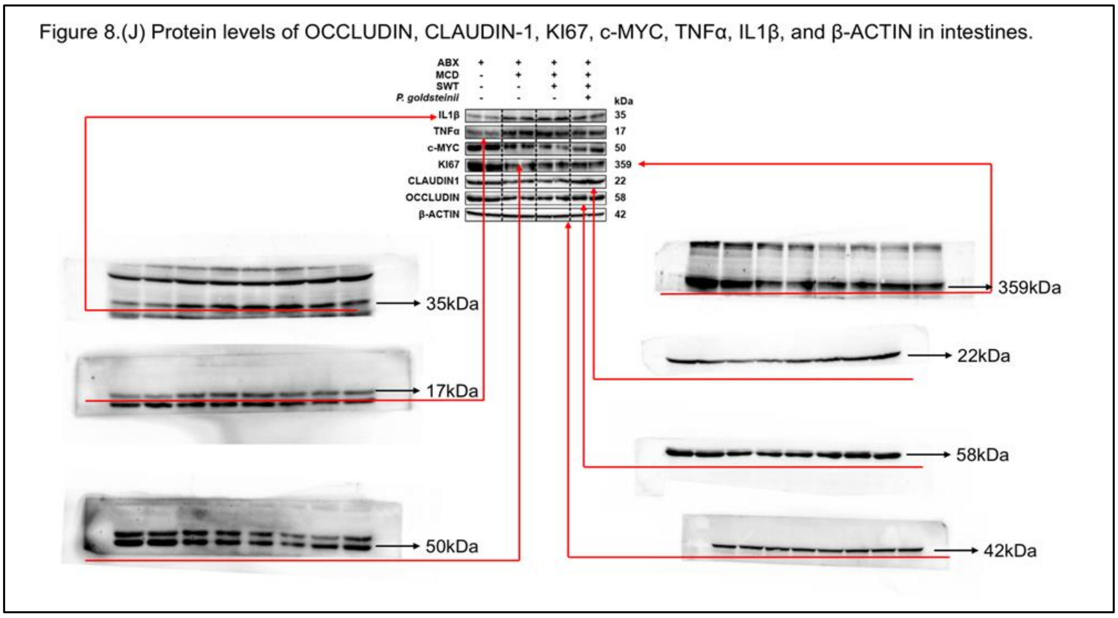

Figure S12. The original picture of Western blot.

3. Supplementary tables

3.1 Supplementary Table 1. The drug materials and their authentication in SWT.

Herbs	Batch number	Medicinal parts	Feature
Rehmannia glutinosa Libosch.	23101301	dry root tuber	thick slices, black and shiny, moist and soft, easy to stick together
Angelica sinensis (Oliv.) Diels	20231029	dry roots	thin sheet, dark yellow, light brown rings on the cross-section
Paeonia lactifora Pall.	24011702	dry roots	thin sheet, slightly brownish red or off-white on the cross-section
Ligusticum chuanxiong Hort.	20230918	dry rhizomes	thin sheet, yellowish-white or grayish-yellow on the cross-section

3.2 Supplementary Table 2. The concentration of main chemical compositions in SWT.

ID	Compound name	CAS No.	m/z	Retention time (min)	Ion mode	Mass Error (ppm)	Peak areas (%)
1	Turanose	547-25-1	365.1055	0.88	POS	0.09	10.89269673
2	Albiflorin	39011-90-0	481.1704	4.56	POS	0.01	8.436918568
3	3-N-butyl-4,5-dihydrophthalide	62006-39-7	193.1223	9.43	POS	0.03	7.509835324
4	Senkyunolide I	94596-28-8	207.1017	5.73	POS	0.48	6.127487043
5	Gentianose	25954-44-3	543.1322	0.81	POS	-0.02	5.175719767
6	5-Hydroxymethylfurfural	67-47-0	127.039	2.53	POS	0.57	2.735099151
7	Paeoniflorin sulfite	1146967-98-7	543.1182	3.97	NEG	0.73	2.59923306
8	Manninotriose	13382-86-0	549.1675	0.81	NEG	0.56	2.360811503
9	Isoferulic acid	25522-33-2	195.0653	5.07	POS	0.78	1.175922387
10	Benzoylalbiflorin	184103-78-4	585.1966	6.64	POS	-0.02	1.034601393
11	Isochlorogenic acid A	2450-53-5	515.1188	5.02	NEG	-1.4	0.90210873
12	Camelliaside A	135095-52-2	755.2029	4.47	NEG	-1.51	0.565185695
13	Rehmannioside D	81720-08-3	731.2267	1.97	NEG	2.2	0.482232941
14	Lactiflorin	1361049-59-3	507.1509	5.51	NEG	0.26	0.330727044
15	Ligustilide	4431-01-0	191.1067	10.17	POS	0.39	0.278528953
16	Levistolide A	88182-33-6	381.206	11.37	POS	-0.14	0.262804094
17	Cnidilide	3674-03-1	195.1381	10.11	POS	0.54	0.14662104
18	Oxypaeoniflorin	39011-91-1	519.1478	4.09	POS	0.92	0.102566394
19	Hesperidin	520-26-3	609.1831	5.21	NEG	0.94	0.094689146
20	Isomartynoside	94410-	651.2	5.76	NEG	1.48	0.040923

ID	Compound name	CAS No.	m/z	Retention time (min)	Ion mode	Mass Error (ppm)	Peak areas (%)
		22-7	304				326
21	Cordycepin	73-03-0	252.1092	1.42	POS	0.36	0.0369108
22	Verbascoside	61276-17-3	647.1946	4.8	POS	-0.15	0.021795575

3.3 Supplementary Table 3. Primers used in qPCR (Mice).

Genes	Forward primer (5′-3′)	Reverse primer (3′-5′)
Acta2	GTCATCCACAGACAGAGTAGG	CTCCCAACAGACCTGTCTATAC
c-Myc	GGAACTATGACCTCGACTAC	CTGCTGTTGCTGGTGATA
Dgat1	CCGATTCTTCCAAGGGAACTAT	ATCGTAGTTGAGCACGTAGTAG
Fasn	TAAAGCATGACCTCGTGATGAA	GAAGTTCAGTGAGGCGTAGTAG
Hprt1	CAGACTTTGTTGGATTTGAAA	GCTCATCTTAGGCTTTGTAT
Il1b	AATCTCACAGCAGCACATC	AGCAGGTTATCATCATCATCC
Mcp1	TTGTAAGTCCAGAGGTTTCTCC	CTGTTGATCTACAGTTTCGCAG
Ocln	TGCTTCATCGCTTCCTTAGTAA	GGGTTCACTCCCATTATGTACA
Plin2	ATGAGTCAAGCCATGGACATG	AAAGAGTGTTCATAGGCGAGAT
Tjp1	CTGGTGAAGTCTCGGAAAAATG	CATCTCTTGCTGCCAAACTATC
Tnfα	GTCCCCAAAGGATGAGAAGT	TTTGCTACGACGTGGGCTAC
